# The Use of Ginger Bioactive Compounds in Pregnancy: An Evidence Scan and Umbrella Review of Existing Meta-Analyses

**DOI:** 10.1016/j.advnut.2024.100308

**Published:** 2024-09-28

**Authors:** Kendra A Tiani, Cristina M Arenaz, Maureen K Spill, Margaret J Foster, Julie S Davis, Regan L Bailey, Martha S Field, Patrick J Stover, Amanda J MacFarlane

**Affiliations:** 1 Institute for Advancing Health Through Agriculture, Texas A&M University, College Station, TX, United States; 2 Texas A&M Agriculture, Food and Nutrition Evidence Center, Fort Worth, TX, United States; 3 Center for Systematic Reviews and Evidence Syntheses, Texas A&M University, College Station, TX, United States; 4 Department of Nutrition, Texas A&M University, College Station, TX, United States; 5 Division of Nutritional Sciences, Cornell University, Ithaca, NY, United States; 6 Department of Biochemistry and Biophysics, Texas A&M University, College Station, TX, United States

**Keywords:** ginger, bioactive compounds, nausea, vomiting, pregnancy

## Abstract

Ginger is a commonly used nonpharmacological treatment of pregnancy-related symptoms including nausea and vomiting, inflammation, and gastrointestinal dysfunction. Determining the efficacy of ginger is particularly important during pregnancy and lactation when maternal and neonatal detrimental effects may be a concern. This evidence scan and umbrella review aimed to assess the extent and quality of the evidence regarding the effectiveness and safety of using dietary preparations of ginger during pregnancy and lactation. We searched MEDLINE, Embase, CAB Abstracts, and International Pharmaceutical Abstracts up to 20 December, 2023, to identify maternal and neonatal outcomes associated with ginger use during pregnancy or lactation compared to placebo or conventional medicines. Outcomes for which a meta-analysis (MA) of intervention studies was identified were synthesized in an umbrella review. The AMSTAR-2 (A MeaSurement Tool to Assess systematic Reviews-2) tool was used to critically appraise the reviews. The percent overlap in primary studies was calculated overall and pairwise for each included MA. Data extracted from each MA included the summary estimate of the effect of ginger, the formulation of the ginger treatment, gestational timepoint at intervention, population enrolled in the study, type of intervention, comparator intervention, and number of study participants. The evidence scan identified 90 articles relevant to ginger use during pregnancy and lactation. Seven MAs of ginger use for treating nausea and vomiting of pregnancy reported 22 independent studies with a 49% study overlap overall. The majority of the MAs found a significant positive effect of ginger on the improvement of nausea in pregnancy compared with placebo, or equivalence to conventional treatments, and no evidence of significant adverse effects. The quality of the MAs ranged from critically low to low. The evidence suggests that ginger is effective at reducing nausea in pregnancy; however, the included studies contained substantial heterogeneity and were of low quality.

**Statements of significance**

Despite common use of dietary ginger preparations among pregnant populations, recent umbrella reviews of ginger use have not focused on the potential health outcomes of ginger consumption in this vulnerable population.

## Introduction

Ginger (*Zingiber officinale Roscoe*) is a perennial herb of the Zingiberaceae family originating from southeast Asia. The ginger rhizome (commonly referred to as root) is used worldwide as a culinary spice in food preparation and cultural food rituals and as a medicinal supplement to prevent or mitigate a diverse range of pathologies in humans. In the past decade, ginger has been evaluated for the treatment of gastrointestinal dysfunction, nausea and vomiting, inflammation, type 2 diabetes mellitus and insulin resistance, and as a galactagogue [[1], [2], [3]]. Additionally, clinical trials have been conducted showing improvements in nausea-related quality of life of chemotherapy patients, demonstrating further populations that may benefit from ginger use [4]. The use of ginger to reduce side effects in chemotherapy has been widely studied, but no clear mechanism of action has been determined. In pregnancy, ginger is often consumed because of its purported antiemetic properties and potential to treat common diseases such as colds, headaches, muscle aches, and nausea [3]. Ginger has also been used historically in southeast Asia as a natural galactagogue to promote lactation; however, the potential mechanism as well as evidence of an effect is not clear [5]. Because of the prevalence of ginger use during pregnancy and lactation in supplements as well as food and drink, evidence for the safety and efficacy of the use of ginger is a priority [[6], [7], [8]].

The bioactive components of ginger include terpenoids, phenolics, gingerols, shogaols, zingiberene, and zingerone but the concentrations of each individual component is influenced by the type and variety of ginger as well as its preparation [1]. The mechanism of the potential therapeutic effect of ginger is not fully understood and may vary by the outcome of interest. Gingerol analogs are potential candidates for the gastrointestinal therapeutic properties of ginger potentially through increased gastrointestinal motility and spontaneous peristaltic activity [9]. Ginger use is associated with reduced tachygastric activity induced by circular vection [10] and had a beneficial effect on reducing slow-wave dysrhythmias due to hyperglycemia [11]. Additionally, the suppression of serotonin 5-HT3 receptors has been shown *in vitro*, which may be in part responsible for the antiemetic effect of ginger [12]. Both animal models and human clinical trials have also demonstrated modulating effects of ginger on inflammation [[13], [14], [15]]. The anti-inflammatory effect of ginger may be because of the inhibitory effects on the PI3K/Akt and Nf-kB pathways, as well as COX enzymes and 5-lipoxogenase [16,17]. In animal studies, ginger extract has been shown to induce hypoglycemia [14], and human clinical trials in individuals with type 2 diabetes mellitus and gestational diabetes mellitus have shown improvements in fasting blood glucose levels and related outcomes [18,19].

Although numerous systematic reviews and MAs have focused on the use of ginger in human health outcomes, an umbrella review focused on pregnancy and lactation-related outcomes is lacking [5,20]. The primary research questions addressed in this review were as follows: *1)* What is the extent and quality of the existing evidence regarding the effectiveness and safety of the use of oral ginger preparations during pregnancy and lactation? And *2)* What are the major maternal and fetal outcomes of oral ginger use during pregnancy and lactation? The secondary research question was as follows: What is the extent of the overlap in primary studies included in the published MAs and how does that influence the reliability of the conclusions? The objective of this umbrella review was to evaluate the methodological quality and scientific rigor of the included reviews with a focus on developing guidelines for the use of ginger during pregnancy across populations worldwide.

## Methods

### Protocol and registration

This review followed the established PRISMA extension protocol for scoping reviews, PRISMA-ScR [21]. The PRISMA checklist is available in Supplemental Table 1. The methodological direction on summarizing systematic reviews was taken from Aromataris et al. [22] of the Joanna Briggs Institute. The protocol was registered at OSF Registries (Open Science Framework, Center for Open Science, osf.io/registries) using the Generalized Systematic Review Registration Form [23].

### Information sources and search strategy

A preliminary evidence scan was performed to identify all maternal and neonatal outcomes associated with oral ginger use during pregnancy and lactation. This consisted of a broad systematic literature search performed across MEDLINE, Embase, CAB Abstracts, and International Pharmaceutical Abstracts databases to include all articles up to 20 December, 2023. The full search strategy was guided by an experienced research librarian (MF) on the Ovid platform and is available in Supplemental Table 2. A broad initial search strategy was employed to capture the full scope of the literature in an evidence scan, which was then used to identify the major outcomes associated with the use of oral ginger preparations during pregnancy and lactation.

### Selection of sources of evidence

Covidence systematic review software (Veritas Health Innovation, 2024) was used to import and remove duplicate results of the systematic literature search as well as to screen selected studies for eligibility. The de-duplicated articles were screened by title and abstract for inclusion on the basis of the selection criteria for the evidence scan. The full-text of the articles that passed initial title and abstract screening was retrieved and evaluated for eligibility on the basis of the same selection criteria. All excluded full-text references are reported in Supplemental Table 3, including the reason for exclusion. All screening was completed in parallel by ≥2 reviewers and disagreements about inclusion were resolved by a third reviewer (JD, MF, KT, or CA).

### Selection criteria for the evidence scan

The population, intervention/exposure, comparison, outcomes (PICO) framework was used to establish the inclusion and exclusion criteria for this review [24]. All full-text peer-reviewed articles in the English language up to December 20, 2023, were eligible for inclusion.

#### Inclusion criteria for evidence scan

The following inclusion criteria were used for the evidence scan: *1*) Population: healthy pregnant or lactating individuals not receiving treatment of any disease or undergoing surgery, *2*) Intervention: the oral administration of a ginger root (rhizome) dietary preparation in the form of a supplement, extract, powder, or similar at a defined dose ingested in the form of a capsule, pill, drink, or other orally ingested medium (intervention will be hereafter referred to as “ginger”), *3*) Control or comparator: control was placebo or no intervention, and comparators were nonpharmacological treatments including vitamin B6, antiemetics, or acupressure, and *4*) Outcomes assessed: maternal, fetal, neonatal, or infant/child health outcomes and adverse effects associated with use of ginger preparations.

#### Exclusion criteria for evidence scan

The following exclusion criteria were used for the evidence scan: *1*) Not pregnant or lactating individuals, *2*) route of ginger administration besides oral, *3*) exclusive clinical population undergoing surgery or other treatment, *4*) no health outcomes assessed, and *5*) preclinical study or *in vitro* study design.

### Data extraction for the evidence scan

Following full-text screening, the selected 90 studies were extracted for basic relevant information by 2 independent researchers (KT and JD) using a predetermined extraction template in Covidence. The extracted data for all references included title, author, date of publication, journal, abstract, type of study (review or primary study), and health outcomes. For primary studies, the type of primary study was recorded as either randomized controlled trials (RCTs), nonrandomized intervention studies, longitudinal cohort studies, nested case-control studies, case-control studies, cross-sectional studies, or specified other type of study. For review articles, the type of review [narrative review, meta-analysis (MA), scoping review, systematic review, umbrella review or other review], number of included articles, and type of studies included (RCTs, nonrandomized intervention studies, longitudinal cohort studies, nested case-control studies, case-control studies, cross-sectional studies, or other studies) was recorded. All study types were extracted for the primary health outcomes grouped by maternal health outcomes (including lactation outcomes), birth and neonatal health outcomes, and infant and child health outcomes. The extraction form included maternal health outcome subcategories of adverse effects, anemia, fertility, gestational weight gain, health behaviors, healthcare utilization, human milk composition and quantity, immune function, micronutrient status, mode of delivery, morbidity, mortality, postpartum weight loss, risk of gestational diabetes, risk of hypertensive disorders during pregnancy, and weight status. The birth and neonatal health outcome categories were birth weight, fetal death, gestational age, head circumference, length of hospital stay, mid-upper arm circumference, NICU admission, small/large for gestational age, stillbirth, and neonatal mortality. The infant and child health outcome categories were anemia, developmental milestones including neurocognitive development, growth, healthcare utilization, morbidity, mortality, risk of child food allergies and atopic diseases, and risk of childhood metabolic disorders including diabetes. Additionally, there was a designated space on the extraction form for reviewers to fill in other health outcomes and information. The primary studies identified in the evidence scan were additionally extracted for total participants per group, study population, study site information, gestational age, intervention, comparator(s), treatment duration, outcomes, and funding sources.

### Data selection and extraction for the umbrella review

The evidence scan was used to identify the primary outcomes reported in all relevant sources. The type of study design and the outcomes of ginger preparation use during pregnancy and lactation were tallied to inform the primary outcome(s) of interest to be evaluated in the umbrella review(s). Further selection criteria were applied to the evidence scan sources to screen for inclusion in the umbrella review(s) on the basis of the prioritized outcome(s). These inclusion criteria were as follows: *1*) MA study design, *2*) published within the last 10 y, and *3*) focused on the identified primary outcome(s). Screening was completed independently by 2 researchers (KT and CA).

Additional data were extracted from the MAs included in the umbrella review. This included study population characteristics, total participants in each group, treatment and dose, comparator and dose, outcomes, summary statistics [odds ratio (OR), standardized mean difference (SMD), weighted mean difference (WMD), mean difference (MD), or risk ratio (RR) and confidence intervals], treatment duration, and funding sources. Studies that included multiple intervention groups were disaggregated to evaluate the single pairwise intervention groups for oral ginger preparation intervention compared with control or oral ginger preparation intervention compared with comparator. The included primary studies of each MA were additionally extracted for study type, total participants per group, study population, study site information, gestational age, intervention, comparator(s), treatment duration, outcomes, and funding sources. Extractions for the umbrella review were completed by KT and reviewed for quality by CA, and any disagreements were resolved by consensus.

### Critical appraisal assessment

The included MAs were critically reviewed for quality and potential bias using the AMSTAR-2 (A MeaSurement Tool to Assess systematic Reviews-2) tool [25]. The tool is a checklist of 16 items that can be categorized into 6 major domains. Items 1–3 of the checklist cover the review planning stage; items 4–7 cover the execution of the review including methods for the search strategy, screening, and extractions; item 8 requires adequate descriptions of the included studies to be available; items 9, 10, 13, and 14 consider if adequate risk of bias (RoB) and heterogeneity investigations of the included primary studies have been conducted; and items 11, 12, and 15 assess if a valid quantitative synthesis method was used for the MA including adequate investigation of publication bias. Finally, item 16 covers disclosures of funding or other potential conflicts of interest. The AMSTAR-2 assessment was completed independently by 2 researchers (KT and CA), and a consensus was reached by discussion.

### Synthesis of the results of the evidence scan and umbrella review

**FIGURE 1:**
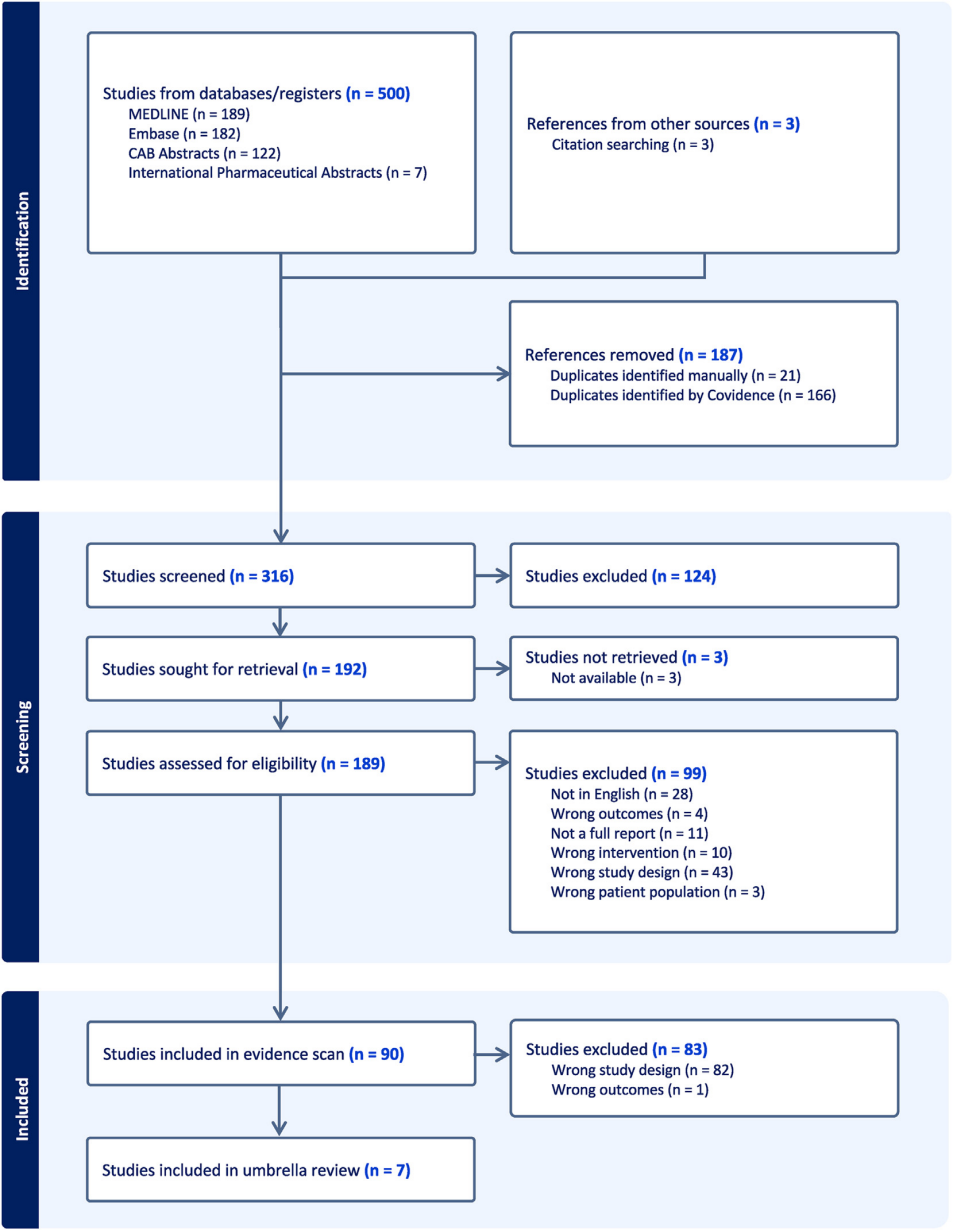
PRISMA flow diagram. Flowchart of the identification and selection of studies included in the broad evidence scan and umbrella review of meta-analyses.

A PRISMA flow diagram was used to show the stages of the evidence scan and umbrella review (Figure 1). Study characteristics and results are described narratively, summarized in tables, and, where applicable, presented as graphs (GraphPad Prism 10, GraphPad Software). The amount of overlap of included RCTs in the MAs was displayed descriptively as a citation matrix. Percent overlap was calculated between each individual review and across all included MAs overall using the corrected covered area (CCA) formula: $CCA=\frac{N-r}{\left(r\times c\right)-r}$ where *N* is the total included primary studies across all reviews including overlap, *r* is the number of individual primary studies, and *c* is the number of reviews.

## Results

### Selection of sources of evidence

#### Evidence scan

**FIGURE 2:**
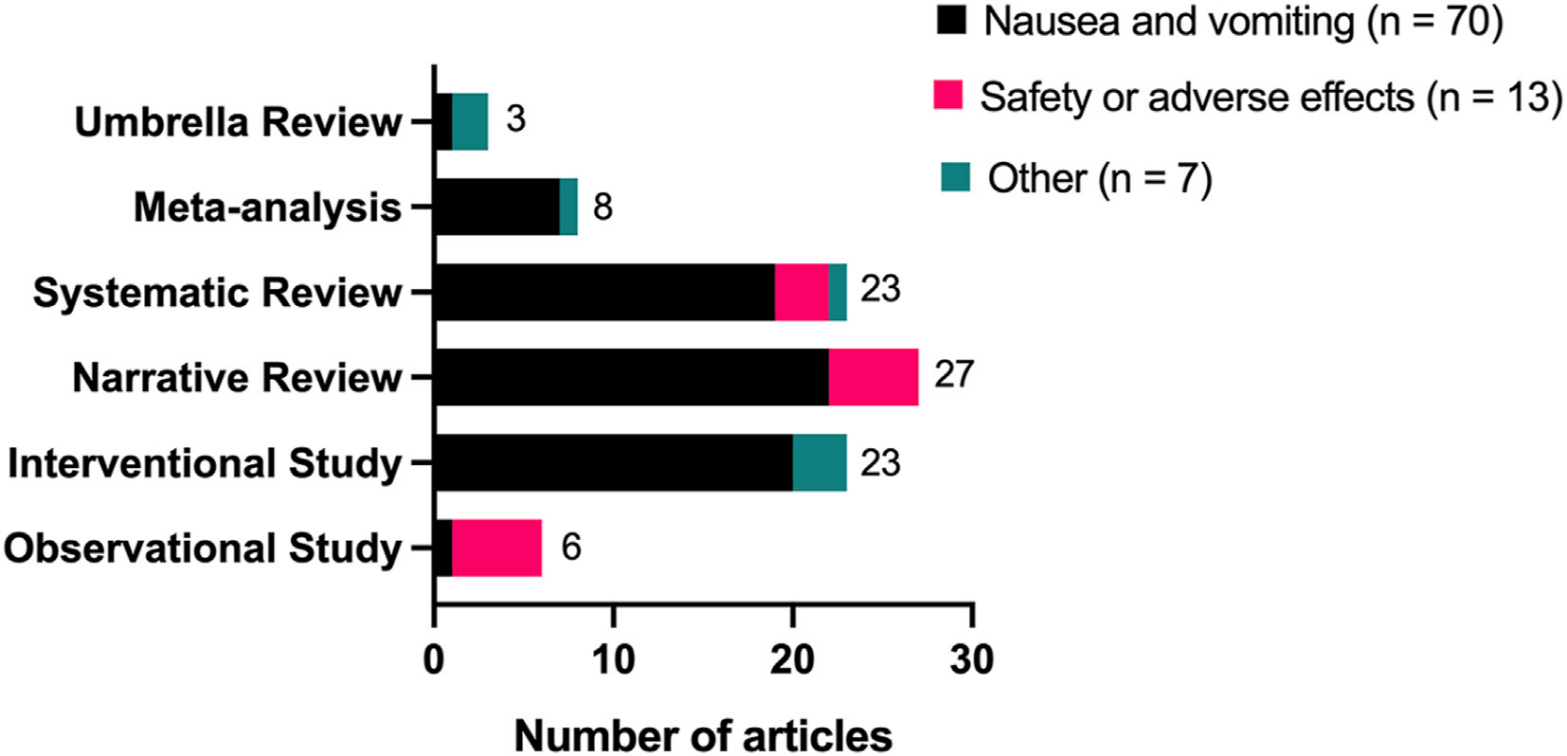
Primary outcomes of interest identified in the evidence scan presented by study type. The majority of studies included in the evidence scan focused on the outcome of nausea and vomiting or on the safety or adverse effects of ginger use during pregnancy. Studies categorized as “other” encompass outcomes investigating lactation, gestational diabetes, and other various human health related outcomes.

The final search was conducted on 20 December, 2023. The detailed search strategy is available in Supplemental Table 2. The citations of the references identified in the evidence scan were manually searched for additional relevant literature to be screened. Figure 1 contains the PRISMA flowchart summarizing the systematic literature search and selection of included studies for the evidence scan and umbrella review. In total, the search strategy and citation searching identified 316 unique results that were screened for relevance and eligibility. Relevant title and abstract screening resulted in a total of 192 studies, 189 of which were successfully retrieved for full-text screening. After full-text screening, 99 sources were excluded because they were not full reports (abstract only or full-text not available), were not written in English, or involved the wrong study population, intervention, outcomes, or study design. The detailed reference list with the reason for exclusion is available in Supplemental Table 3. In total, 90 references matched our inclusion and exclusion criteria for the evidence scan and were extracted for relevant information including type of study and primary outcomes (Supplemental Table 4). The characteristics of the articles included in the evidence scan are presented descriptively and in a summary table (Table 1). The results of the evidence scan are presented descriptively and as a graph with the types of included studies categorized by the primary outcome of interest (Figure 2). Additional characteristics of the interventional and observational primary studies included in the evidence scan are available in Supplemental Table 4.

**TABLE 1:** General characteristics of articles included in the evidence scan.

Characteristic	Articles, *n*	Percentage^1^
Publication year	(*n* = 90)	
<2000	4	4.4
2000–2009	29	32.2
2010–2019	40	44.4
2020–December 2023	17	18.9
		
Publication type	(*n* = 90)	
Review article	61	67.8
Primary study	29	32.2
		
Type of review	(*n* = 61)	
Narrative review	27	44.3
Systematic review	23	37.7
Meta-analysis	8	13.1
Umbrella review	3	4.9
		
Type of primary study	(*n* = 29)	
Observational	6	20.7
Interventional	23	79.3

1 Values displayed are rounded to one decimal point, the original values sum to 100%.

#### Umbrella review of meta-analyses

Studies identified in the evidence scan were further screened to identify MAs of RCTs examining the effect of oral ginger preparation intake, resulting in 6 included MAs and 1 included network MA on the outcome of nausea and vomiting of pregnancy (NVP). An umbrella review of these MAs was initiated. These MAs reviewed 22 unique human interventional studies on the use of ginger preparations to improve NVP. There were 19 cited primary interventional studies that were common to the evidence scan as well as the included MAs. A summary table of the individual characteristics of all interventional studies cited by MAs in the umbrella review is available in Supplemental Table 5. The results of the umbrella review are presented descriptively in a table of study characteristics (Table 2) and a summary table of the results including effect estimates of the outcomes (Table 3) [20,[26], [27], [28], [29], [30], [31]].

**TABLE 2:** Characteristics of meta-analyses included in umbrella review.

Author year	Studies, *n*	Participants, *n* (I/P/C)	Gestational age (wk)	Ginger intervention dose^1^	Comparator (s)^2^ (dose)	Duration	AMSTAR-2 analysis	Funding or CoI
Tan et al. [20], 2023	14 (RCT)	1436 (655/354/427)	<20	500–2500 mg/d	Placebo, vitamin B6 (40–160 mg/d), dimenhydrinate (100 mg/d), metoclopramide (30 mg/d)	≥4 d (4–21 d)	Critically low	None
Gaur et al. [26], 2022	7 (RCT)	763 (379/NA/384)	<20	Ginger (1000–1950 mg/d)	Placebo and vitamin B6 (30–160 mg/d)	≥3 d (3–21 d)	Low	None
Hu et al. [27], 2020	13 (RCT)	1191 (551/223/417)	<20	Ginger (750–2500 mg/d)	Placebo and vitamin B6 (30–160 mg/d)	≥3 d (3–21 d)	Critically low	NFSC grant no. 81602852
Sridharan et al. [28], 2018	19 (RCT)	1854 (845/325/684)	<20	Ginger (450–2500 mg/d)	Placebo, vitamin B6 (30–160 mg/d), dimenhydrinate (100 mg/d), metoclopramide (30 mg/d), chamomile 500 mg/d, pyridoxine and doxylamine (20–30 mg/d)	≥3 d (3–60 d)	Low	None
Matthews et al. [29], 2015	16 (RCT)	1641 (753/310/578)	<20	Ginger (450–2500 mg/d)	Placebo, vitamin B6 (30–75 mg/d), dimenhydrinate (100 mg/d), metoclopramide (30 mg/d), chamomile (500 mg/d), pyridoxine, and doxylamine (20–30 mg/d)	≥3 d (3–21 d)	Low	University of Liverpool; Health Research Board, Ireland; Chochrane Fellowship, National Institute for Health Research, UK
Viljoen et al. [30], 2014	12 (RCT)	1178 (574/221/383)	<18	Ginger (500–2500 mg/d)	Placebo, vitamin B6 (30–75 mg/d), metoclopramide (30 mg/d), and dimenhydrinate (100 mg/d)	≥4 d (4–21 d)	Critically low	None
Thomson et al. [31], 2014	6 (RCT)	508 (256/252/NA)	<20	Ginger (1000–2500 mg/d)	Placebo and vitamin B6 (75 mg/d)	≥4 d (4–21 d)	Critically low	None

Abbreviations: AMSTAR-2, A MeaSurement Tool to Assess systematic Reviews; CoI, conflicts of interest; C, comparator (not including placebo); I, intervention; NA, not applicable; P, placebo.

1 Ginger root (rhizome) interventions included preparations of powdered ginger, ginger extracts, or brewed ginger.

2 Comparators were either placebo, vitamin B6, dimenhydrinate, metoclopramide, pyridoxine and doxylamine, or chamomile.

**TABLE 3:** Results summary of the included meta-analyses.

Author year	Method (fixed or random)	Effect estimate vs. placebo	Effect estimate vs. conventional treatments	Summary of results
Tan et al. [20], 2023	Random-effects model	NVP effective rate: RR: 1.68; (1.09, 2.57); *P* = 0.0018; *I*^2^ = 76.4% Nausea score: WMD: –1.21 (–2.34, –0.08); *P* = 0.036; *I*^2^ = 66.0% Vomiting score: WMD: 0.05 (–0.23, 0.32); *P* = 0.743; *I*^2^ = 0%	NVP score: WMD: := –0.52 (–0.79, –0.24); *P* ≤ 0.001; *I*^2^ = 0% (included metoclopramide) Vomiting score: SMD: 0.30 (–0.12, 0.73); *P* = 0.160; *I*^2^ = 51.1%	Ginger had a higher effective rate and improved nausea symptoms compared with placebo, but vomiting was not statistically significant. Ginger was more effective than vitamin B6 or metoclopramide on NVP score across 2 trials but had no effect on vomiting compared with vitamin B6.
Gaur et al. [26], 2022	Random-effects model	n/a	NVP overall: SMD: 0.36 (–0.21, 0.60); *P* = 0.02; *I*^2^ = 17% Nausea score: SMD: –0.15 (–0.28, 0.05); *P* = 0.87; *I*^2^ = 50.0% Vomiting score: SMD: –0.05 (–0.11, 0.21); *P* = 0.57; *I*^2^ = 0%	Both vitamin B6 and ginger treatment improved NVP symptoms overall across treatment duration in 2 studies; however, vitamin B6 may be more effective because of a greater reduction in NVP scores overall in the MA. There was no difference in nausea or vomiting scores between vitamin B6 and ginger.
Hu et al. [27], 2020	Random-effects and fixed-effects models	Fixed-effects NVP improvement ratio: OR: 7.475 (4.133, 13.520); *P* < 0.001; *I*^2^ = 30.1% Fixed-effects nausea score: SMD: 0.821 (0.585, 1.056); *P* < 0.001; *I*^2^ = 38.9% Random-effects vomiting score: SMD: 0.549 (0.585, –0.268); *P* = 0.188; *I*^2^ = 91.4%	Random-effects NVP: pooled OR: 1.239 (0.495, 3.102); *P* = 0.647; *I*^2^ = 57.3% Random-effects nausea score: SMD: 0.199 (–0.102, 0.500); *P* = 0.196; *I*^2^ = 65.7% Random-effects vomiting score: SMD: 0.331 (–0.145, 0.808); *P* = 0.173; *I*^2^ = 85.9%	Ginger was more effective for NVP symptoms overall and severity of nausea compared with placebo, but no effect was found for vomiting score. Compared with vitamin B6, no results significantly favored ginger or vitamin B6.
Sridharan et al. [28], 2018	Random-effects model	Nausea scores: direct comparison WMD: –4.2 (–6.5, –1.9); *P* ≤ 0.05; mixed treatment comparison WMD: –4.7 (–6.0, –3.4); *P* ≤ 0.05 Vomiting control: pooled OR: 34.9 (3.9, 316.20); *P* ≤ 0.05	Nausea scores: direct comparison WMD: –0.1 (–0.3, 0.1); *P* > 0.05; mixed treatment comparison WMD = –0.1 (–0.3, 0.1); *P* > 0.05	Ginger has therapeutic benefits in the treatment of NVP including decreasing nausea scores and improving vomiting control. However, this was a network MA, and results may change with head-to-head clinical trials. Minimal inconsistency was reported between direct and mixed comparisons.
Matthews et al. [29], 2015	Random-effects and fixed-effects models	n/a	Fixed-effects NVP score day 3: SMD: 0.0 (–0.25, 0.25); *P* = 0.99; *I*^2^ = 0% Random-effects no symptom improvement: average RR: 0.84 (0.47, 1.52); *P* = 0.57; *I*^2^ = 51.85%	Evidence for ginger over placebo was limited and no MA was conducted; however, individual studies show benefit of ginger over placebo. No difference was shown between vitamin B6 and ginger NVP symptom scores on treatment day 3 or for the number of patients reporting no improvement in symptoms.
Viljoen et al. [30], 2014	Random-effects and fixed-effects models	Fixed-effects nausea scores: MD: 1.20 (0.56, 1.84); *P* = 0.0002; *I*^2^ = 0% Random-effects vomiting scores: MD: 0.72 (–0.03, 1.46); *P* = 0.06; *I*^2^ = 71%	Random-effects nausea scores: MD: 0.34 (–1.52, 2.20); *P* = 0.72; *I*^2^ = 91% Random-effects vomiting scores: MD: –0.07 (–0.48, 0.35); *P* = 0.76; *I*^2^ = 44%	Ginger significantly improved nausea scores compared with placebo and was not significant for reducing vomiting. Ginger did not reduce nausea or vomiting symptoms compared with vitamin B6.
Thomson et al. [31], 2014	Random-effects model	NVP overall: pooled OR: 4.89 (1.88, 12.73); Q-statistic = 33.72	n/a	Ginger treatment was more effective than comparators on reducing NVP symptoms overall. However, a vitamin B6 comparison study was included in the placebo comparison which may have diluted the effect of ginger compared with placebo.

Abbreviations: MA, meta-analysis; MD, mean difference; NVP, nausea and vomiting of pregnancy; OR, odds ratio; RR, risk ratio; SMD, standardized mean difference; WMD, weighted mean difference.

### Characteristics of sources of evidence

A total of 90 sources published in peer-reviewed journals were included in the evidence scan focused on the use of ginger preparations during pregnancy (Supplemental Table 4). This evidence scan was not restricted by study design type or by maternal, fetal, neonatal, or infant outcomes of ginger preparation use during pregnancy and lactation. The major characteristics of the sources included in the evidence scan are summarized in Table 1. The evidence scan identified 29 relevant primary studies and 61 relevant review articles that were published between 1991 and December, 2023, and included primary studies conducted in 13 different countries. The 29 identified primary studies included 6 observational studies and 23 interventional studies. Out of the 61 identified review articles, there were 27 narrative review articles, 23 systematic reviews, 8 MAs, and 3 umbrella reviews (Table 1). The major outcomes of ginger preparation use during pregnancy investigated in the literature were as follows: safety and adverse outcomes, lactation-related outcomes, gestational diabetes outcomes, and treatment of nausea and vomiting (Figure 2).

#### Characteristics of observational studies

In the primary literature, there were 6 identified observational studies that included case-control studies [32,33], longitudinal cohort studies [[34], [35], [36]], and an observational clinical feasibility study [37]. The studies were conducted on participants in South Korea, Norway, Italy, Ethiopia, Belgium, and Canada. Overall, the observational studies were primarily focused on studying the safety of oral ginger preparations during pregnancy and documenting any adverse effects (Figure 2), although evaluating the efficacy of ginger preparations in treating NVP was a common secondary outcome [35,37], and evaluating determinants of hyperemesis gravidarum was the primary focus of 1 observational study [32]. The number of participants who were exposed to ginger during pregnancy varied from 9 to 1052 across all observational studies (Supplemental Table 4).

#### Characteristics of interventional studies

The evidence scan also identified 23 interventional studies that were conducted to compare an oral ginger preparation intervention to either placebo, vitamin B6, acupressure, or the antiemetic drugs metoclopramide or dimenhydrinate. Out of these interventional studies, 21 were RCTs and 2 were nonrandomized intervention studies [38,39]. There were 15 trials conducted comparing a ginger preparation arm to a placebo or no intervention arm, 8 trials comparing a ginger preparation to vitamin B6, 2 trials comparing a ginger preparation intervention to a conventional antiemetic medication, and 1 study comparing a ginger preparation to P6 acupressure. The range of ginger preparation intervention and comparator doses were as follows: ginger preparation between 450 mg/d and 2500 mg/d, vitamin B6 between 30 mg/d and 160 mg/d, metoclopramide at 30 mg/d, and dimenhydrinate at 100 mg/d. The most common outcome investigated was the treatment of NVP, which was the focus of 20 articles (Figure 2, Supplemental Table 4). The remaining studies were a trial examining lactation-related outcomes of a ginger preparation [40] and 2 trials investigating outcomes of a ginger preparation intervention on control of blood glucose levels in women with gestational diabetes [19,41] (Figure 2). The total number of participants analyzed in each interventional study ranged from 21 to 235 participants with an average of 88 participants across all study arms. The majority of the included interventional studies were conducted in Iran [19,39,[41], [42], [43], [44], [45], [46], [47], [48], [49], [50]], Thailand [40,[51], [52], [53], [54]], and Australia [55,56], whereas the 4 remaining studies were conducted in Pakistan [57], Indonesia [38], Denmark [58], and the United States [59]. A nonrandomized intervention study on oral ginger preparation use and NVP outcomes by Moghadam et al. [39] was the only intervention study discovered in the evidence scan that was not also included in an MA. This exclusion was likely because of a lack of information on randomization in the study methods, as well as the absence of a control or placebo arm in this intervention study. Four additional intervention studies were discovered through citation searching of the included umbrella reviews, MAs, and systematic reviews and underwent full-text screening. As a result, 1 nonrandomized intervention study was screened and included [38]. Supplemental Table 5 contains the extraction information on the total of 19 relevant English full-text intervention studies. In addition, 3 abstracts that were excluded because of availability or language are noted for reference.

#### Characteristics of narrative and systematic reviews

Most of the narrative and systematic reviews focused on the primary outcome of NVP, but several were primarily focused on safety and adverse outcomes of ginger preparation use in pregnancy (Figure 2). From the total of 27 included narrative reviews, 22 focused on NVP and 5 focused on safety and adverse outcomes of use of ginger preparations. From the total of 23 systematic reviews that were included, 19 focused on the primary outcome of maternal NVP (Figure 2). Overall, 13 of the 19 NVP systematic reviews summarized RCTs, and 6 of the 19 NVP systematic reviews included RCTs as well as observational studies, nonrandomized intervention studies, case reports, or other systematic reviews. A total of 3 systematic reviews out of the overall 23 were conducted on the safety and adverse effects of ginger use during pregnancy and lactation [6,60,61]. These 3 reviews included RCTs, cross-sectional studies, and longitudinal cohort studies. Finally, a single systematic review of RCTs was conducted on the lactation-related outcomes of maternal oral ginger preparation use; however, there was only 1 included RCT in this systematic review that used a ginger intervention that was not combined with any other herbal medicine [5,40].

#### Characteristics of meta-analyses

A total of 8 MAs were identified in the evidence scan. There was a single Cochrane MA that focused on lactation-related outcomes of the use of oral galactagogues [62]; however, an MA for the galactagogue effect of ginger was not able to be conducted because of a lack of RCTs with an intervention of oral ginger that was not combined with any other herbal medicines. There were no MAs conducted on the effect of ginger preparation use on gestational diabetes-related outcomes such as control of blood glucose concentrations. There were 6 MAs [20,26,27,[29], [30], [31]] and 1 network MA [28] of oral ginger use focused on the outcome of maternal NVP (Figure 2). Table 2 summarizes the key characteristics of the included MAs. There were varying quantities and formulations of ginger preparations used in the intervention, but the most common treatment was 1000 mg/d of encapsulated dried ginger root. The outcomes of interest were improvement of maternal NVP, as well as documentation of any adverse effects or safety concerns. The number of participants in the MAs ranged from 129 to 1436 across all primary study arms including a ginger intervention and comparator or placebo arms, whereas a network MA by Sridharan et al. [28] contained 1854 participants in total. The study settings of the component RCTs were regional hospitals, clinics, or health centers. Detailed PICO information on the RCTs included in the MAs is available in Supplemental Table 5. The comparison groups in 6 out of the 7 MAs were a ginger preparation intervention compared with placebo or a comparator, whereas 1 MA focused on comparing the effectiveness of ginger to a vitamin B6 intervention [26].

#### Characteristics of umbrella reviews

Three existing umbrella reviews on human health outcomes of ginger use were identified in the evidence scan; however, none were focused exclusively on a pregnant population, or maternal nausea and vomiting specifically of pregnancy [[63], [64], [65]]. The umbrella review by Zhang et al. [65] focused broadly on human health outcomes and discussed 3 MAs included in this review, which focused on ginger for treating NVP [27,30,31]. The umbrella review by Crichton et al. [63] also focused broadly on oral ginger consumption and human health outcomes, but included 5 systematic reviews, a narrative review, an MA, and an umbrella review [1,5,6,9,27,47,66]. The umbrella review by Li et al. [64] in 2023 focused more specifically on the use of ginger for treating nausea and vomiting of any etiology and included 5 MAs that focused on pregnant women [20,26,27,30,31].

### Evidence scan results

The maternal health outcomes of ginger preparation use identified in the evidence scan were NVP, safety and/or adverse effects of ginger use, control of blood glucose levels in gestational diabetes patients, and human milk quantity and quality.

#### Nausea and vomiting of pregnancy

The treatment of NVP was the most investigated outcome of ginger use in pregnancy across all included articles. A case-control study by Ashebir et al. [32] found that women with hyperemesis gravidarum were less likely to have used ginger of undefined preparation during pregnancy. An observational clinical feasibility study by Laekeman et al. [37] also found that ≥60% of patients were satisfied with the effect of a 50 mg ginger ethanolic extract tablet [drug extract ratio (DER) of 10:1] on reducing NVP symptoms. A prospective comparative study by Portnoi et al. [35] of ginger product exposure during pregnancy noted a mild positive effect of ginger on treating NVP; however, the primary focus of most identified observational studies was the investigation of adverse effects.

There were 12 interventional studies identified in the evidence scan, which compared a ginger intervention with a placebo or no intervention arm and all authors found that ginger preparations were effective in reducing ≥1 symptom of NVP including the frequency or severity of nausea, vomiting, or retching [38,42,44,[46], [47], [48], [49], [50],^54^,^56^,^58^,^59^]. Data for nausea showed the most evidence of a positive effect, whereas the data for vomiting were not consistent. Additionally, there were 8 RCTs that compared ginger to a vitamin B6 intervention arm, which is a conventional treatment of NVP, and 7 of these found that ginger preparations were equally effective at reducing the symptoms of NVP compared with vitamin B6 [44,45,50,51,53,55,57]. The remaining RCT found that 1000 mg/d of dried ginger was more effective at treating nausea and equally effective at treating vomiting compared with the conventional treatment of 40 mg/d of vitamin B6 [43]. There were 2 RCTs that compared a ginger preparation intervention with pharmacological antiemetics (dimenhydrinate and metoclopramide) [46,52]. The authors found no difference between the intervention groups in terms of nausea scores, and additionally, Pongrojpaw et al. [52] showed no difference in vomiting scores of 100 mg/d of dimenhydrinate compared with 1000 mg/d of encapsulated dried ginger.

Li et al. [64] recently conducted an umbrella review of ginger for treating nausea and vomiting of any etiology and included 5 MAs on NVP [20,26,26,30,31]. The authors concluded that despite low methodological quality of the included primary studies, ginger has potential in treating NVP [64]. In 2022, Zhang et al. [65] conducted an umbrella review of systematic reviews and MAs on the health outcomes of *Zingiberaceae* plants and curcumin, and included 3 MAs focused on NVP [27,30,31]. Zhang et al. [65] reported a positive effect of ginger on easing symptoms of pregnancy discomfort, but they concluded that 2 included reviews by Hu et al. [27] and Viljoen et al. [30] failed to show an effect of ginger on the improvement of NVP. However, the authors did not discuss the difference between placebo and vitamin B6 comparators on the results, a significant factor because of the established role of vitamin B6 as a conventional treatment of NVP. An additional umbrella review by Crichton et al. [63] examined oral ginger consumption on multiple human health outcomes and included systematic reviews of ginger use during pregnancy as well as a single MA [27]. Crichton et al. [63] also concluded that ginger was safe and effective at reducing nausea incidence and severity compared with placebo in pregnant women.

Additionally, 7 MAs were included in the evidence scan, which examined the effect of use of ginger preparations on the maternal outcome of NVP. These reviews are analyzed in greater detail in the umbrella review below.

#### Adverse effects

The potential safety or adverse effects of the use of oral ginger preparations or oral ginger intake during pregnancy was the primary focus of 6 included observational studies (Figure 2). Three of these observational studies reported on potential adverse effects. Choi et al. [33] conducted a case-control study within the Korean Motherisk Program of mothers who used naturopathic dried ginger at a dose of 0.3–7200 mg/d compared with age-matched mothers who were not exposed to dried ginger during pregnancy. Concerningly, the authors saw a marginally increased risk of stillbirth [OR: 7.8 (0.9–70.3); *P* = 0.05], but no increased risk of major malformations [OR: 4.9 (0.9–25.5); *P* = 0.051] or other fetal and neonatal adverse effects (*P* > 0.05). However, a dose-dependent effect was not investigated across the large range of ginger doses, and the effect of additional herbal medication cannot be excluded. Additionally, Trabace et al. [36] analyzed a retrospective cohort of mothers in the South of Italy and found an association with shorter gestational age (*P* = 0.034) and a smaller newborn skull circumference (*P* = 0.001) with oral ginger herbal product intake compared with nonusers of ginger products or users of other herbal medicines. There was no significant effect on birth weight. However, the relevant sample size of this study was quite small because only 9 interviewed mothers out of 630 reported using ginger products during pregnancy, and the type of ginger preparation or dosages consumed were not reported by the authors. On the other hand, Portnoi et al. [35] did not find a significant increase in the rates of major malformations because of ginger product exposure in a prospective cohort of 187 Canadian women who called the Motherisk Program. Additionally, Heitmann et al. [34] analyzed a large longitudinal cohort of 68,522 Norwegian women, 1020 of whom reported using ginger (ginger herbal products, supplements, or alternative/herbal remedies) while pregnant. Of those 1020 women, 466 used ginger during the first trimester and found no increased risk of any reported pregnancy outcome associated with the use of ginger [34]. The strengths of this study were the large sample size of exposed women, the prospective longitudinal study design, and the inclusion of potential confounding factors in the analysis. However, none of these observational studies reported complete information on the source, composition, and dosage of the ginger product exposures. More recently, Laekeman et al. [37] conducted a prospective, interventional, open clinical feasibility study with the intent of detecting potential maternal complications or neonatal malformations in 44 pregnant patients exposed to ≤100 mg/d of a concentrated ethanolic extract of ginger root (EXT.GR10) starting at <12 wk of gestation. One 50 mg dry EXT.GR10 ginger extract tablet was ≅500 mg dry ginger root powder (DER of 10:1). The authors found no relationship between number of tablets taken and any adverse maternal or neonatal events [37]. In the RCTs and interventional studies, there were infrequently reported minor side effects in the ginger group including heartburn, stomachache, or indigestion [44,57]. However, no major maternal adverse effects were reported, and no fetal abnormalities or adverse outcomes were reported that rose above baseline and could be attributed to the ginger intervention. Adverse effects of dietary ingredients, supplements, or products may be collected by means other than peer-reviewed literature, and thus may not be reported in the studies included in this review.

#### Gestational diabetes mellitus

Two randomized clinical trials examined the maternal outcome of control of blood glucose levels in gestational diabetes mellitus patients. Hajimoosayi et al. [19] found that a 6-wk intervention of 1500 mg/d ginger herbal tablets (Vomigone, Dineh Company) led to a significantly greater reduction in fasted blood glucose levels compared with baseline in the ginger treatment group after the intervention period (*n* = 37; *P* = 0.004) compared with the placebo group that did not show a significant reduction in blood glucose levels (*n* = 33). Fasting insulin levels and the Homeostasis Model Assessment index were also significantly reduced by the ginger intervention compared with placebo. However, an RCT by Bahramian et al. [41], which included 75 participants, did not see a significant reduction of fasted blood glucose or hemoglobin A1C levels after 8 wk on the ginger capsule intervention (1000 mg/d dried ginger root), but did see significant improvements in postprandial blood glucose levels. Because of the conflicting results of these clinical trials, more rigorous clinical trials with larger sample sizes are needed to evaluate the efficacy of ginger in treating the symptoms of gestational diabetes mellitus.

#### Lactation

A single randomized double-blind controlled trial was conducted to investigate the effect of ginger capsules (1000 mg/d dried ginger root) on breast milk volume [40]. Paritakul et al. [40] found that the ginger group had a greater breast milk volume on day 3 [MD: 56.0 mL/d; (20.9, 91.0); *P* < 0.01; *n* = 33 placebo; *n* = 30 ginger] but not on day 7 [MD: –9.8 mL/h (–69.5, 49.8); *P* = 0.24; *n* = 21 placebo; *n* = 15 ginger] of the study. However, there was a significant loss to follow up by day 7, and 1 h pumped milk volume was used for quantification, unlike 24 h breast milk volume quantification used on day 3 postpartum. As a result of the small trial size and these limitations, more research is needed to form conclusions on the effect of a ginger intervention to increase breast milk volume.

### Umbrella review results

**FIGURE 3:**
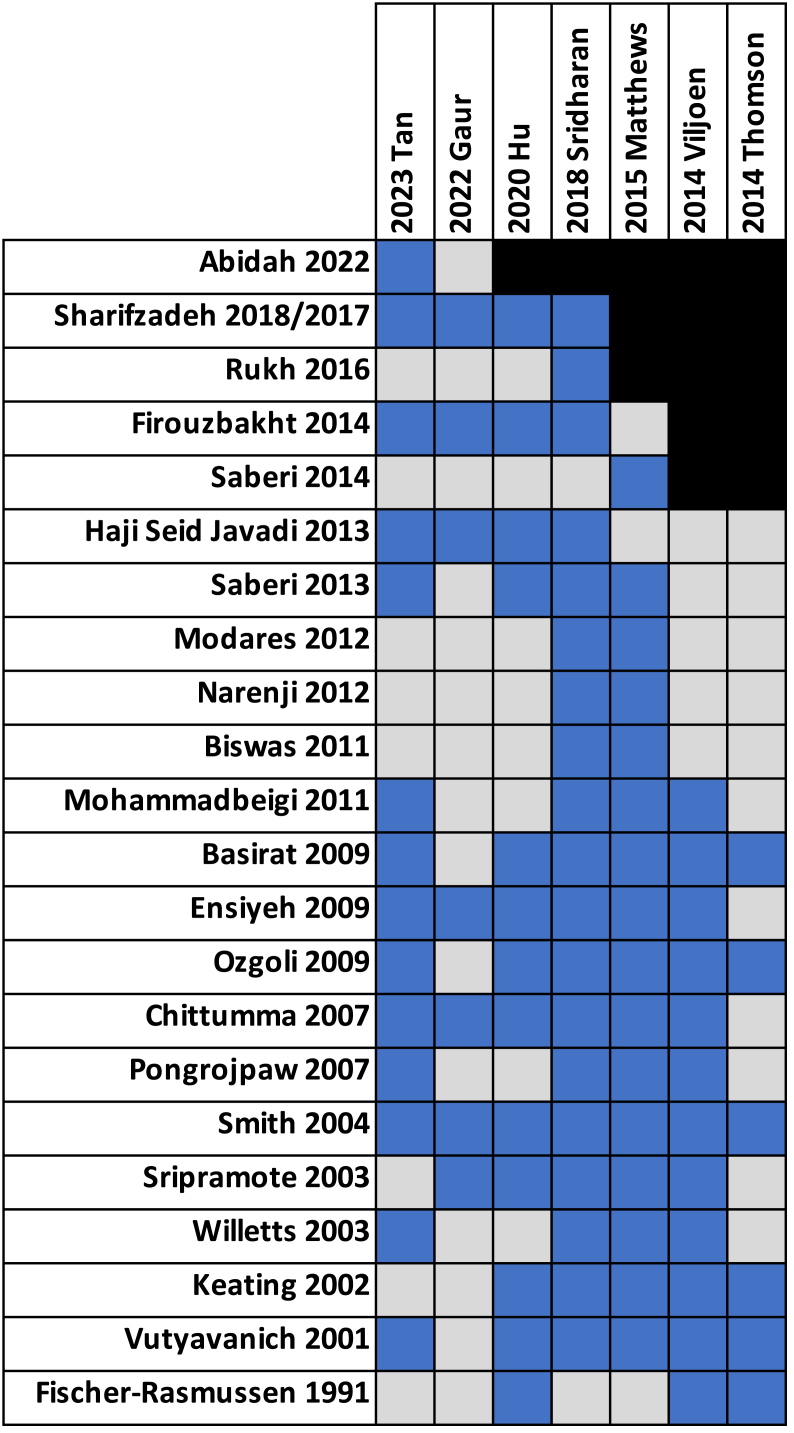
Citation matrix of the overlap in cited intervention studies between meta-analyses. The blue squares represent inclusion, the gray squares represent studies that were not cited, and black square shows studies not eligible for inclusion according to year of publication of meta-analysis.

Because of the quantity of available MAs on ginger use during pregnancy, an umbrella review and synthesis of the results was warranted. The major outcomes reported across the MAs were severity of nausea, frequency of vomiting, or severity of NVP. Outcome effect size was variably reported as either ORs, MD, WMD, or SMD. Both fixed-effects and random-effects models were used in the quantitative synthesis and estimates of heterogeneity between studies varied widely from 0% to 91% (Table 3) [20,[26], [27], [28], [29], [30], [31]]. Publication bias was difficult to assess as all the included MAs had <10 RCTs per quantitative synthesis, meaning that publication bias is unclear across the included reviews despite the statistical methods of testing employed by some authors. The individual intervention studies and RCTs cited in each MA are displayed in a citation matrix (Figure 3). A table of the populations, interventions, comparators, outcomes assessed, study site information and location, and sources of funding of the component RCTs can be found in Supplemental Table 5. The intervention across all studies was ginger, and the comparators included in MAs were either placebo, vitamin B6, or conventional medicine.

#### Critical appraisal assessment

The AMSTAR-2 tool was used to critically appraise the methodological quality of the included MAs on NVP. The overall ratings ranged from critically low (57.1%) to low (42.9%) (Table 2). However, to better understand the overall ratings, the questionnaire items were grouped into 5 categories that included review planning, search strategy, screening, and extraction, descriptive analysis, RoB and heterogeneity, MA methodologies, and conflicts of interest. In the review planning category (items 1–3), all reviews had a majority of positive ratings (yes or partial yes). All reviews included the components of PICO in their research questions and inclusion criteria (item 1). However, 28.6% of reviews did not have an established protocol prior to conducting the review (item 2) and a single review did not adequately justify their choice of including only RCTs (item 3). In the search, screening, and extraction category (items 4–7), the majority of the ratings were positive; however, 57.1% of studies were only a partial yes for use of a comprehensive search strategy (item 4) and 85.7% of studies did not provide a list of excluded articles with justification (item 7). All reviews performed study selection in duplicate (item 5), and all but 1 review reported performing extractions in duplicate (item 6). All reviews adequately described the included primary studies (item 8), although 71.4% of reviews were missing details on the study setting and population. In the RoB and heterogeneity category (items 9, 10, 13, and 14), all authors adequately assessed RoB using a Cochrane RoB tool (item 9); however, no authors reported on potential conflicts of interest or the source of funding for the component studies of the MAs (item 10). All reviews accounted for RoB when discussing the results (item 13), and 85.7% reviews adequately investigated heterogeneity as well as discussed potential causes and effects on the interpretation of the results of the MA (item 14). All reviews used appropriate methods for statistical combination of results and reported using random-effects models when applicable to deal with clinical heterogeneity in the data (item 11). However, 57.1% of MAs did not investigate the effects of individual study RoB on the results of the MA through sensitivity analyses (item 12) and did not adequately investigate or discuss publication bias in the interpretation of their results (item 15). Finally, all review authors reported funding sources or potential conflicts of interest for their MA (item 16). A summary table of the AMSTAR-2 critical appraisal is available in Supplemental Figure 1.

#### Results of individual sources of evidence

Tan et al. [20] recently conducted an MA on NVP, which included summaries of 14 relevant RCTs on the effect of a ginger preparation intervention on NVP. The authors performed an MA of ginger preparations compared with placebo looking at the visual analog scale (VAS) rating for nausea, number of vomiting episodes, effective rate, and adverse events as well as ginger compared with conventional medicine looking at the Rhodes index of nausea and vomiting and number of vomiting episodes. The effective rate was defined as the proportion of patients who had significantly improved symptoms or had recovered. Ginger had a higher effective rate on NVP compared with placebo across 3 RCTs of 196 participants [RR: 1.68 (1.09, 2.57); *P* = 0.018; *I*^2^ = 76.4%] [42,47,54] and showed decreased severity of nausea as measured by VAS in 3 RCTs of 181 participants [WMD: –1.21 (–2.34, –0.08); *P* = 0.036; *I*^2^ = 66.0%] [42,44,54]. However, there was no difference on the frequency of vomiting compared with placebo in 2 trials of 114 patients [WMD: 0.05 (–0.23, 0.32); *P* = 0.743; *I*^2^ = 0%] [42,44]. Significant heterogeneity was present in the comparisons of the effective rate and nausea scores for ginger preparation interventions compared with placebo, and a GRADE analysis conducted across all analyzed outcomes rated the quality of evidence as low. Ginger preparations were also compared with “conventional medicine” in 2 trials of 122 patients and an MA showed that ginger was more effective at improving the Rhodes index of nausea and vomiting [WMD: –0.52 (–0.79, –0.24); *P* ≤ 0.001; *I*^2^ = 0%; GRADE: moderate] compared with vitamin B6 or metoclopramide [46,50]. No effect of ginger on vomiting instance compared with vitamin B6 was found in a random-effects MA of 3 trials with 182 patients [43,44,50] [SMD: 0.30 (–0.12, 0.73); *P* = 0.160; *I*^2^ = 51.1%; GRADE: low]. Six of the included studies in this MA, with a total of 420 patients, reported on the potential adverse effects of ginger compared with placebo. The reported side effects were stomachache and heartburn; however, no difference was found in the reported adverse effects for the ginger preparations compared with placebo [RR: 1.57 (0.63, 3.91); *P* = 0.336; GRADE: very low].

Gaur et al. [26] conducted an MA examining the efficacy of ginger preparations compared with vitamin B6 and used random-effects models to quantitatively analyze 7 RCTs. The overall change in nausea and vomiting score between ginger and vitamin B6 was analyzed in 2 studies, which both showed an improvement in NVP symptoms overall [45,51]. The vitamin B6 intervention was significantly more effective than ginger, although both groups showed improvement with vitamin B6 or ginger treatment compared with baseline. The SMD was 0.36 (–0.21, 0.60), *P* = 0.02, with no significant heterogeneity between RCTs (*I*^2^ = 17%). After removing high-heterogeneity studies, there was no difference in the nausea score alone between groups in 5 RCTs [44,50,51,53,55] and no difference in the vomiting score between groups in 4 RCTs [43,44,50,53]. The SMD in nausea scores between groups was –0.15 (–0.28, 0.05), *P* = 0.87, with significant heterogeneity remaining (*I*^2^ = 50.0%). The SMD in vomiting scores was –0.05 (–0.11, 0.21), *P* = 0.57, with no remaining heterogeneity (*I*^2^ = 0%). In this MA, ginger preparations were found to be an effective treatment of nausea or vomiting because of no significant difference in the effect compared with an established therapeutic. For NVP symptoms overall, there was evidence that vitamin B6 was more effective than ginger; however, there were only 2 included RCTs in this analysis with 218 participants in total, and a high risk of performance bias, detection bias, or reporting bias. Adverse effects were not included in the scope of this MA although the authors noted no congenital malformations, abnormal pregnancies, or delivery outcomes as a result of the ginger intervention. Stomachache, heartburn, and dizziness were reported as minor side effects of the ginger preparations.

An MA by Hu et al. [27] used a fixed-effects model to investigate the ratio of symptom improvement in 5 RCTs [42,47,54,58,59] and found that ginger preparation interventions were more effective at improving NVP symptoms compared with placebo [OR: 7.475 (4.133, 13.520); *P* < 0.001]. No significant heterogeneity (*I*^2^ = 30.1%) or publication bias was detected. The ginger interventions also had a significant effect on reducing the severity of nausea compared with placebo [SMD: 0.821 (0.585, 1.056); *P* = 0.000; *I*^2^ = 38.9%] across 5 RCTs [42,44,49,50,54], which were analyzed using a fixed-effect model. The instance of vomiting compared with placebo was not significant among 5 RCTs [42,44,49,50,54] when analyzed using a random-effects model, but significant heterogeneity in studies was found [SMD: 0.549 (0.585, –0.268); *P* = 0.188; *I*^2^ = 91.4%]. Meta regression showed that the location, duration, gestational stage, intervention dose, and outcome measure of the component RCTs had no significant effect on heterogeneity; however, a leave-one-out analysis found that Firouzbakht et al. [44] was the main contributor to study heterogeneity, and the summary effect estimate 95% confidence interval was significant after its removal [SMD: 0.883 (0.225, 1.541), *I*^2^ = 83.8%]. Additionally, a random-effects model used to investigate the effectiveness of ginger compared with vitamin B6 on ratio of NVP symptom improvement in 2 RCTs [43,55] showed a greater effect of ginger, but this was not statistically significant [pooled OR: 1.239 (0.495, 3.102); *P* = 0.647; *I*^2^ = 57.3%]. However, the evidence was weak because of a lack of high-quality RCTs, significant heterogeneity between RCTs, and unclear publication bias. There was no significant effect of ginger compared with vitamin B6 on reducing the severity of nausea [SMD: 0.199 (–0.102, 0.500); *P* = 0.196; *I*^2^ = 65.7%] or vomiting [SMD: 0.331 (–0.145, 0.808); *P* = 0.173; *I*^2^ = 85.9%] across 6 RCTs using a random-effects model [[43], [44], [45],^50^,^51^,^53^]. Significant heterogeneity in the data was present for both estimates. A leave-one-out analysis of nausea scores in the ginger group compared with vitamin B6 reduced heterogeneity to *I*^2^ = 34.2% and reversed the estimate of the effect to favor vitamin B6 [SMD: 0.324 (0.126, 0.532)] after removing 1 RCT [53]. A leave-one-out analysis did not change the significance of the effect for vomiting compared with vitamin B6. No obvious publication bias was observed. Adverse effects were not the focus of this MA; however, the authors noted that there were no reported congenital abnormalities or abnormal pregnancy and delivery outcomes because of the ginger preparation intervention. There were some reports of dizziness, stomachache, and heartburn as a result of ginger consumption.

Sridharan et al. [28] conducted a network MA and trial sequential analysis on the primary outcome of nausea scores across 19 RCTs with a ginger preparation intervention. Decreased nausea scores were reported for ginger compared with placebo [direct comparison WMD: –4.2 (–6.5, –1.9); *P* ≤ 0.05; mixed treatment comparison WMD: –4.7 (–6.0, –3.4); *P* ≤ 0.05] and vitamin B6 compared with placebo [direct comparison WMD: –3.7 (–6.9, –0.5); *P* ≤ 0.05; mixed treatment comparison WMD: –4.1 (–6.0, –2.2); *P* ≤ 0.05]. There was no significant difference for ginger compared with vitamin B6 [direct comparison WMD: –0.1 (–0.3, 0.1); *P* > 0.05; mixed treatment comparison WMD: –0.1 (–0.3, 0.1); *P* > 0.05]. Both direct and mixed treatment comparisons were estimated using a random-effects model. There was minimal inconsistency reported between direct and mixed comparisons (score of 1–1.5); however, the causes of this inconsistency were not further discussed. The authors concluded that there was only moderate evidence to support the use of ginger in treating NVP, and all other treatments were considered to have very low-quality evidence. No publication bias was observed for the ginger compared with placebo and the ginger compared with vitamin B6 comparisons. A trial sequential analysis was conducted for ginger compared with placebo and adequate evidence was available to confirm the effectiveness of ginger. The evaluation of adverse events was a secondary outcome of this review. In their network MA, the authors found that the ginger and vitamin B6 interventions were associated with fewer incidences of adverse events compared with placebo, although the definition of an adverse event was not stated.

Matthews et al. [29] conducted a Cochrane systematic review and MA of NVP interventions including oral ginger preparation interventions. A total of 16 RCTs were included; however, few MAs were conducted that contained multiple RCTs, and no RCTs were able to be combined in a quantitative analysis for the comparison of ginger with placebo because of differing outcome measures. In the ginger compared with vitamin B6 comparison, only 2 outcomes were quantitatively analyzed. These outcomes were nausea and vomiting score on treatment day 3 and no improvement in symptoms. No difference in nausea and vomiting scores was detected between groups on day 3 in an fixed-effects MA of 2 RCTs with 251 participants [SMD: 0.0 (–0.25, 0.25); *P* = 0.99; *I*^2^ = 0%] [41,53], and no heterogeneity or subgroup differences were found. The RR of the number of women who reported no improvement in symptoms was not different between ginger and vitamin B6 in a random-effects analysis of 2 RCTs that included 360 participants [average RR: 0.84 (0.47, 1.52); *P* = 0.57; *I*^2^ = 51.85%] [43,55]. However, moderate heterogeneity in studies was present. The evaluation of adverse effects was a primary focus of this MA; however, few included studies reported on maternal and fetal adverse outcomes. Of the trials that did report data on side effects and adverse events, all were underpowered to detect significant differences in adverse events between groups [29].

Viljoen et al. [30] conducted a systematic review and MA of 12 RCTs with 1178 participants total. A Cochrane RoB assessment concluded that all RCTs had moderate-to-high RoB. However, because of the differences in outcome measures, not all studies could be quantitatively analyzed. Seven studies compared a ginger preparation to placebo. Two RCTs reported an improvement in nausea symptoms by a change in VAS scores and showed that ginger significantly decreased symptoms compared with placebo [MD: 1.20 (0.56, 1.84); *P* = 0.0002; *I*^2^ = 0%] in a fixed-effects analysis with no subgroup differences based on ginger dosage [42,54]. Another 2 RCTs reported on the number of women who showed an improvement in nausea symptoms by VAS scores and found no difference in the reduction of symptoms in the ginger group compared with placebo [RR: 2.00 (0.77, 5.19); *P* = 0.15; *I*^2^ = 59%] [47,59]. The heterogeneity was moderate, but no subgroup differences in the duration of intervention were present. The remaining 3 studies that compared ginger with placebo were not meta-analyzed; however, 1 RCT showed an improvement in nausea severity with ginger treatment [46] and 1 crossover RCT found a significant decrease in combined symptom relief scores in the ginger treatment group compared with placebo [58]. The remaining RCT examining ginger compared with placebo did not report values for a treatment effect, only graphical results [56]. All included studies reported that ginger reduced vomiting episodes compared with placebo; however, the effect estimates were not all sufficient for quantitative synthesis and an MA was only able to be conducted between 2 RCTs [42,54]. This MA showed a nonsignificant reduction in vomiting episodes with ginger compared with placebo [MD: 0.72 (–0.03, 1.46); *P* = 0.06; *I*^2^ = 71%]. A comparison of ginger preparation interventions to vitamin B6 included 4 individual RCTs [43,51,53,55]. Two RCTs were combined in an MA of the improvement in nausea symptoms [43,53] and 3 studies were pooled in an MA of the reduction of vomiting [43,53,55]. Ginger did not significantly decrease VAS of nausea compared with vitamin B6 in a random-effects model [MD: 0.34 (–1.52, 2.20); *P* = 0.72; *I*^2^ = 91%]; however, differing dosages may have contributed to heterogeneity. Also, ginger did not significantly reduce the number of vomiting episodes compared with vitamin B6 [MD: –0.07 (–0.48, 0.35); *P* = 0.76; *I*^2^ = 44%] in a random-effects MA with moderate heterogeneity and no significant subgroup differences. Ginger preparations were also compared with conventional antiemetics dimenhydrinate or metoclopramide in 2 separate studies that could not be meta-analyzed but showed no difference between groups and equal efficacy of ginger on the treatment of NVP symptoms [46,52]. Reporting on adverse effects of ginger was a secondary objective of this review. The authors noted no significant difference between the ginger and placebo, and ginger and vitamin B6 interventions for most major or minor reported adverse events or side effects. There was an increased risk of belching in the ginger intervention group compared with vitamin B6.

An MA by Thomson et al. [31] was conducted in 2014 and included 6 RCTs that were deemed to be of satisfactory quality according to a Cochrane RoB assessment [42,47,54,55,58,59]. Five of these RCTs compared a ginger preparation intervention to placebo; however, a major limitation of this review was the inclusion of an RCT by Smith et al. [55], which used vitamin B6 as the comparator instead of a placebo. Because vitamin B6 is a commonly prescribed treatment of NVP, it should not be considered a placebo and may decrease the estimate of the effect of ginger preparations. Despite this, a random-effects model demonstrated that ginger was more effective than the comparators at improving symptoms of NVP [pooled OR: 4.89 (1.88, 12.73)] with significant heterogeneity (Cochrane Q-statistic: 33.72; df = 5; *P* < 0.0001). Publication bias was not evaluated. Adverse effects were not the focus of this review; however, the authors noted that common side effects of the ginger interventions were reflux, heartburn, gastric discomfort.

#### Overlap assessment

There were 22 individual RCTs or intervention studies cited across the 7 MAs. A citation matrix was generated to showcase which individual RCTs were cited in each MA, although not all RCTs were included in every quantitative synthesis conducted in the overarching MA (Figure 3). This is because of different outcomes assessed, which could not be combined or removal of individual RCTs because of high heterogeneity within the MA. The number of duplicate primary studies among each of the included MAs was reported for each MA as the percent overlap (Supplemental Figure 2). The CCA for all reviews was 49% and there was a high level of agreement in the review conclusions. This is likely because of the similar scope in topics between reviews including similar outcomes, populations, and interventions assessed. However, these results of the MAs must be interpreted with caution because high levels of overlap overall and between specific quantitative analyses can lead a single trial or multiple trials to have excess influence on the results of the umbrella review because of nonindependence of the included primary studies. The overlap in primary studies between specific MA comparisons is reported in the synthesis of the Results section.

#### Synthesis of results of umbrella review

The MAs included in the umbrella review did not evaluate all relevant RCTs in their quantitative synthesis because of the heterogeneity of interventions including differing ginger dosages, preparations, comparators, length of intervention, and outcome measurement. The RoB assessments in these studies were variably evaluated by review authors; however, the component RCTs should be considered having moderate-to-high RoB. The primary sources of bias were because of difficulties in personnel and participant blinding of a ginger preparation (e.g., if the included RCT used ginger biscuits or ginger drinks), a high risk of detection bias across all RCTs because of self-reported outcomes, unclear or high selective reporting bias due to lack of prespecified outcomes or missing timepoint data, and the inclusion of dietary counseling, which may improve symptoms independently of ginger interventions. A separate RCT conducted by Zick et al. [67] investigated feasibility of blinding of a ginger capsule intervention and found that participants can correctly distinguish a bottle of capsules but not individual capsules. This means participant blinding is a considerable source for bias if no investigation is conducted to ensure the efficacy of the blinding method, especially in interventions that used biscuits or extracts in a drink. No authors considered all studies to be low RoB across all categories. Publication bias was inconsistently assessed and reported as not present, but this finding must be interpreted with caution because of the low numbers of included RCTs that may invalidate statistical testing for publication bias. The most common MA categories were ginger preparations compared with placebo and ginger preparations compared with active ingredients including vitamin B6 or pharmaceutical antiemetics. The primary outcomes evaluated in these comparisons were improvements in general NVP symptoms overall and independent nausea scores or vomiting scores. The summary statistics calculated by reviews that meta-analyzed 2 or more RCTs are evaluated below.

#### NVP symptoms overall compared with placebo

Tan et al. [20], Hu et al. [27], and Thomson et al. [31] reported results for the improvement in NVP symptoms overall and the effective rate of ginger preparations on NVP symptoms overall compared with placebo. Tan et al. [20] showed that out of 196 participants ginger was more effective than placebo [RR: 1.68, (1.09, 2.57); *P* = 0.0018; *I*^2^ = 76.4%] [42,47,54]. Hu et al. [27] found ginger to be more effective than placebo at improving NVP symptoms across 5 RCTs [42,47,54,58,59], [OR: 7.475 (4.133, 13.520); *P* < 0.001; *I*^2^ = 30.1%]. Thomson et al. [31] analyzed 6 RCTs including 1 vitamin B6 comparator RCT [42,47,54,55,58,59] and still found that ginger was more effective than the comparators at improving NVP [pooled OR: 4.89 (1.88, 12.73)] with significant heterogeneity (Cochrane Q-statistic: 33.72; df = 5; *P* < 0.0001). There was a high level of overlap in primary RCTs between these 3 MAs (CCA = 53.3%) [68,69]. A reduction in overall NVP symptoms was significant in all 3 reviews but this is not surprising because of the high levels of overlap between component primary studies.

#### Nausea scores compared with placebo

Tan et al. [20], Hu et al. [27], Viljoen et al. [30], and Sridharan et al. [28] all reported on nausea scores compared with a placebo across 7 included RCTs. Tan et al. [20] analyzed 3 RCTs with 181 participants in total [42,44,54] and found decreased severity of nausea [WMD: –1.21 (–2.34, –0.08); *P* = 0.036; *I*^2^ = 66.0%]. Hu et al. [27] reported a significant effect on reducing the severity of nausea of the ginger preparations compared with placebo in 5 RCTs [42,44,49,50,54] [SMD: 0.821 (0.585, 1.056); *P* = 0.000; *I*^2^ = 38.9%]. Viljoen et al. [30] showed an improvement in nausea symptoms with a ginger preparation intervention [MD: 1.20 (0.56, 1.84); *P* = 0.0002; *I*^2^ = 0%] across 2 RCTs [42,54], but showed no improvement in the number of women reporting improved nausea symptoms with ginger compared with placebo [RR: 2.00 (0.77, 5.19); *P* = 0.15; *I*^2^ = 59%] between another 2 RCTs [47,59]. Not including the network MA by Sridharan et al. [28], which did not directly report included RCTs, there is still a high level of overlap in primary studies (CCA = 27.7%). Sridharan et al. [28] showed that ginger decreased nausea scores compared with placebo in both a direct and mixed treatment comparison in their network MA [direct comparison WMD: –4.2 (–6.5, –1.9); *P* ≤ 0.05; mixed treatment comparison WMD: –4.7 (–6.0, –3.4); *P* ≤ 0.05]. All 4 MAs showed a reduction in nausea scores with ginger treatment compared with placebo and only 1 MA of 2 RCTs found no difference in the number of patients who reported improvement in nausea scores.

#### Vomiting scores compared with placebo

Tan et al. [20], Hu et al. [27], and Viljoen et al. [30] all quantitatively analyzed vomiting scores with ginger preparation interventions compared with placebo in direct MAs, which included 5 individual RCTs in total. Tan et al. [20] found no difference on vomiting frequency in a direct MA of 2 RCTs with 114 patients [WMD: 0.05 (–0.23, 0.32); *P* = 0.743; *I*^2^ = 0%] [42,44]. Hu et al. [27] showed no significant difference in vomiting instance in an MA of 5 trials [42,44,49,50,54] [SMD: 0.549 (0.585, –0.268); *P* = 0.188; *I*^2^ = 91.4%]. Viljoen et al. [30] showed a nonsignificant reduction in vomiting episodes [MD: 0.72 (–0.03, 1.46); *P* = 0.06; *I*^2^ = 71%] with ginger treatment compared with placebo among 2 RCTs [42,54]. The CCA between RCTs across these 3 MAs was 33.3%, which indicates considerable overlap. A fourth review, a network MA by Sridharan et al. [28], reported mixed treatment comparison estimates for a secondary outcome of better vomiting control compared with placebo and found that ginger significantly improved the control of vomiting in 8 studies of 669 participants [pooled OR: 34.9 (3.9, 316.20); *P* ≤ 0.05]; however, the individual RCTs were not cited and Sridharan et al. [28] was omitted from a percent overlap calculation. Overall, 3 out of 4 reviews found no difference in vomiting scores between ginger preparations and placebo.

#### General NVP symptoms compared with vitamin B6 or antiemetics (conventional treatments)

Tan et al. [20], Gaur et al. [26], Hu et al. [27], and Matthews et al. [29] all investigated the effect of ginger preparations compared with vitamin B6 or pharmaceutical antiemetics. Tan et al. [20] quantitatively analyzed 2 trials of 122 patients [46,50] and showed that ginger was more effective at improving the Rhodes index of nausea and vomiting compared with “conventional medicine,” which included vitamin B6 or metoclopramide [WMD: –0.52 (–0.79, –0.24); *P* ≤ 0.001; *I*^2^ = 0%, GRADE: moderate]. Gaur et al. [26] also meta-analyzed NVP symptoms overall across 2 RCTs [45,51] and found that vitamin B6 was more effective than ginger, although both showed improvements in overall symptoms [SMD: 0.36 (–0.21, 0.60); *P* = 0.02; *I*^2^ = 17%]. Hu et al. [27] used a random-effects model to compare the ratio of general NVP symptom improvement of ginger compared with vitamin B6 across 2 studies [43,55] and found no significant effect [OR: 1.239 (0.495, 3.102); *P* = 0.647; *I*^2^ = 57.3%]. A Cochrane review by Matthews et al. [29] found no difference in NVP scores between ginger and vitamin B6 on day 3 of treatment in an analysis of 2 RCTs with 251 participants [SMD: 0.0 (–0.25, 0.25); *P* = 0.99; *I*^2^ = 0%] [51,53]. Additionally, the RR of the number of patients who reported no improvement in symptoms was not different between ginger and vitamin B6 in a random-effects analysis of 2 RCTs of 360 participants [average RR: 0.84 (0.47, 1.52); *P* = 0.57; *I*^2^ = 51.85%] [43,55]. The calculated overlap between these 4 reviews was moderate (CCA = 12.5%), and the results did not favor either ginger or a conventional treatment of NVP because of opposite results between 2 MAs and no effect found across 3 analyses. However, because the conventional treatments of vitamin B6 or antiemetics were not clearly favored over ginger preparations, ginger may be equally as effective as an established treatment of NVP.

#### Nausea scores compared with vitamin B6 or antiemetics (conventional treatments)

Gaur et al. [26], Hu et al. [27], Viljoen et al. [30], and Sridharan et al. [28] examined the influence of ginger preparations compared with vitamin B6 or antiemetics in MAs on the outcome of nausea scores. After removing high-heterogeneity studies, Gaur et al. [26] found that there was no difference in the nausea score between ginger and vitamin B6 in 5 RCTs [44,50,51,53,55] [SMD: –0.15 (–0.28, 0.05); *P* = 0.87; *I*^2^ = 50.0%]. Hu et al. [27] used a random-effects model of 6 RCTs [[43], [44], [45],^50^,^51^,^53^] and found no significant effect of nausea scores in ginger treatment compared with vitamin B6 [SMD: 0.199 (–0.102, 0.500); *P* = 0.196; *I*^2^ = 65.7%]. However, a leave-one-out analysis that removed 1 RCT [53] reduced heterogeneity and reversed the estimate of the effect to favor vitamin B6 [SMD: 0.324 (0.126, 0.532); *I*^2^ = 34.2%]. Viljoen et al. [30] combined 2 RCTs in an MA of the improvement in nausea symptoms by VAS [43,53]. Ginger did not significantly decrease the VAS of nausea compared with vitamin B6 [MD: 0.34 (–1.52, 2.20); *P* = 0.72]; however, heterogeneity in studies was high (*I*^2^ = 91%). Overall, 1 analysis supported B6 over ginger after a leave-one-out analysis, whereas all other MAs showed no difference in ginger on nausea scores compared with the conventional treatment, vitamin B6. Heterogeneity in studies was high and the overlap between direct MAs was high (CCA = 33.3%). A network MA by Sridharan et al. [28] showed no significant difference for ginger compared with vitamin B6 [direct comparison WMD: –0.1 (–0.3, 0.1); *P* > 0.05; mixed treatment comparison WMD: –0.1 (–0.3, 0.1); *P* > 0.05].

#### Vomiting scores compared with vitamin B6 or antiemetics (conventional treatments)

Tan et al. [20], Gaur et al. [26], Hu et al. [27], and Viljoen et al. [30] evaluated the effect of ginger preparations compared with conventional treatments on vomiting scores across 7 individual RCTs that compared a ginger preparation intervention to vitamin B6. Tan et al. [20] showed that there was no effect of ginger on vomiting instance compared with vitamin B6 [random-effects SMD: 0.30 (–0.12, 0.73); *P* = 0.160; *I*^2^ = 51.1%; GRADE: low] between 3 trials with 182 patients [43,44,50]. Gaur et al. [26] found no difference in vomiting score between ginger and vitamin B6 in a random-effects analysis of 4 RCTs [43,44,50,53] [SMD: –0.05 (–0.11, 0.21); *P* = 0.57; *I*^2^ = 0%]. Hu et al. [27] showed no difference in vomiting scores [SMD: 0.331 (–0.145, 0.808); *P* = 0.173; *I*^2^ = 85.9%] across 6 RCTs [[43], [44], [45],^50^,^51^,^53^] using a random-effects model of ginger compared with “conventional medicine” (vitamin B6), but heterogeneity between studies was high. Viljoen et al. [30] analyzed vomiting scores of ginger treatment compared with vitamin B6 across 3 studies [43,53,55] and found that ginger did decrease vomiting episodes compared with vitamin B6 [MD: –0.07 (–0.48, 0.35); *P* = 0.76; *I*^2^ = 44%]. All 4 reviews that analyzed ginger compared with conventional NVP treatments showed no evidence for a difference in effect of ginger compared with vitamin B6 on vomiting scores in NVP patients. However, there was a high level of overlap between the individual RCTs included in the MAs (CCA = 37.5%).

## Discussion

This evidence scan and umbrella review have a unique perspective compared with the published literature because despite frequent use of ginger preparations among pregnant populations [6,36], recent umbrella reviews of ginger use have not completed an in-depth analysis of the potential health outcomes related to its use in this vulnerable population.

### Evidence scan

The evidence scan identified the major maternal health outcomes associated with ginger use from the peer-reviewed literature: lactation-related effects, treatment of gestational diabetes mellitus, treatment of NVP, and evaluation of adverse effects. The outcomes focused on the improvement of lactation and the treatment of gestational diabetes mellitus symptoms had no significant evidence for an effect of the ginger preparation; however, the level and quality of the evidence was minimal. Adverse effects were investigated across different study methodologies including RCTs, nonrandomized intervention studies, and observational studies. There were some teratogenic concerns related to ginger product use raised as a result of 3 observational studies [33,36,70]. But a further 3 observational studies, including the largest observational study of 1000 participants, did not find any significant adverse effect of ginger use [34,35,37]. However, the interpretation of the results of the observational studies is limited by an inability to determine causality and a higher impact of potential confounding and bias on the results. Finally, no major adverse effects were reported from any of the RCTs or nonrandomized interventional studies included in this evidence scan. Only minor side effects of the ginger preparation interventions were reported including heartburn or indigestion, and the ginger formulation or dose has not been investigated regarding the etiology of gastrointestinal-related side effects. Significant evidence was found to support the safety of ginger use during pregnancy to improve symptoms of NVP.

### Umbrella review

#### Background

The umbrella review was conducted on 7 MAs of the use of ginger to treat NVP, which is most common in early pregnancy and affects ∼50%–80% of all human pregnancies [29,71]. Although commonly referred to as “morning sickness,” symptoms of nausea, vomiting, and retching are typically present throughout the day and may occur individually or concurrently. The most severe presentation of NVP is *hyperemesis gravidarum*, which is not well defined but occurs when persistent NVP leads to significant weight loss and dehydration and may result in hospitalization. In addition to causing substantial maternal discomfort, NVP can have significant social and professional consequences, which makes the validation of safe and effective treatments for NVP a priority [72]. Although there are conventional pharmacological treatments for NVP including antihistamines, pharmaceutical antiemetics are not always recommended for minor NVP [71]. The most commonly prescribed treatment of NVP is vitamin B6 with or without doxylamine, which is recommended by the American College of Obstetricians and Gynecologists (ACOG) as a first-line treatment of NVP (recommendation level A) [71]. Additional level A ACOG recommendations include taking prenatal vitamins prior to fertilization, although the mechanisms by which prenatal vitamins reduce NVP risk are not clear and may be a result of increased vitamin B6 levels or improved nutritional status. Ginger is also commonly used as a nonpharmacological treatment of nausea and vomiting and has a level B recommendation from ACOG for use in treating NVP [35,71].

#### Summary of evidence

The evidence of the effectiveness of ginger preparations on NVP is impeded by the quality of individual RCTs. As a result, several MAs have been conducted to synthesize the overall evidence on the effectiveness of ginger on treatment of NVP. The patients in the included studies were all <20 wk of gestation at enrollment and there were almost no significant differences in age and parity between treatment groups (Supplemental Table 5). The patients received a ginger preparation intervention of between 450 and 2500 mg/d, which lasted 4 d in the majority of studies but ranged from 3 to 60 d across all studies.

In terms of the outcomes evaluated, use of ginger preparations compared with a placebo led to a reduction in overall NVP symptoms across 3 MAs that contained data on the effect of ginger on NVP overall [20,27,31,64]. Four MAs reported on the effect of a ginger preparation intervention on nausea scores, all reviews reported a reduction in nausea scores with the ginger intervention compared with a placebo [20,27,28,30]. However, 3 out of 4 MAs that evaluated instances of vomiting in pregnancy compared with a placebo found no effect of a ginger intervention [20,27,28,30]. There was no conclusive evidence of a difference in the effect of ginger compared with vitamin B6 or conventional antiemetics in NVP symptoms overall or on nausea and vomiting scores individually. This adds credibility to the positive effect of ginger on NVP because no differences were found compared with vitamin B6. However, there was a high overlap in included studies among all analyzed individual MA comparisons for the same outcomes. This high level of nonindependence limits any additional confidence in the effect of the intervention on individual outcomes that might be construed with each additional MA. Finally, all MA reported no significant adverse effects. Minor side effects that were variably reported included heartburn, dizziness, and abdominal discomfort.

### Important considerations

Teratogenic risk is a significant barrier to treatment of many women who are experiencing NVP. The availability of a more palatable, nonpharmacological treatment such as vitamin B6 or ginger may be useful for women who would otherwise choose no treatment at all, preferring the debilitating symptoms of NVP over risk to their pregnancy [72,73]. Additionally, ginger is a common spice, frequently used by pregnant women, and there have been only infrequently reported minor side effects of ginger use. There are no published case studies of toxicity or teratogenicity; however, it should be noted that the regulation of dietary ingredients or supplements is not as rigorous as prescription manufacturers, and most studies did not test product quality, amount of ginger, source of ginger, and other germane considerations. Additionally, potential drug interactions with ginger supplements have been proposed, including hypoglycemia from an additive effect of insulin or metformin use and an increased risk of bleeding with aspirin use because of decreased platelet aggregation from thromboxane synthetase inhibition [70]. However, no interventional studies of ginger use reviewed here have reported complications with platelet aggregation or hypoglycemia in pregnant women, and there is no available data on confirmed side effects, only potential interactions. However, an increased risk of postpartum bleeding because of an antiplatelet effect via drug interactions may not be observed in studies because of the low dose of ginger and limited number of subjects [9,70]. As such, additional appropriately powered dose–response studies including information on potential adverse effects of ginger root preparations with verified identity, purity, dose, and composition are warranted to get an accurate picture of potential adverse effects. Care should be taken to discuss indications or contraindications and possible side effects of ginger use during pregnancy, as well as alternatives such as vitamin B6. There is no established upper intake limit for ginger; however, vitamin B6 has a tolerable upper intake level (UL) of 100 mg/d during pregnancy and lactation [74]. Patients on any dose higher than the UL should be monitored by a physician. Doses of 500 mg/d vitamin B6 have been shown to cause sensory neuropathy and vitamin B6 intakes in excess of the UL can lead to photosensitivity, dermatological lesions, nausea, and heartburn [74]. When deciding on a nonpharmacological treatment of NVP, ginger may be an appealing option because of the evidence of equal efficacy in treating nausea and a lack of documented adverse effects. However, high-quality RCTs are needed to definitively establish equal efficacy.

### Strengths and limitations

A comprehensive search strategy was used to locate and examine all relevant literature in a broad evidence scan which allowed for the review of all types of evidence on the maternal and neonatal health outcomes of ginger use during pregnancy and lactation. Rigorous methods were used to evaluate the evidence including a critical appraisal of included MAs and an analysis of the overlap in individual RCTs between the MAs, which had not been conducted previously for the outcome of NVP. We are confident that no relevant primary studies have been omitted from this review as a result of this overlap assessment and evidence scan.

However, the conclusions of this review are limited by the potential bias of the component studies including poor blinding of participants because of the difficulty of concealing an herbal intervention, self-reporting of outcomes assessed, as well as the variety of different ginger formulations, dosages, and intervention lengths used across studies. A major limitation of this review is the lack of validated matched ginger formulations across intervention studies, which reduces the ability to accurately assess the safety and efficacy of ginger use during pregnancy. Of note, the interventions used in the majority of the RCTs were fresh dried ginger root (rhizome), Zintoma capsules with dry ginger root powder, or unspecified dried ginger. However, 4 studies used a ginger extract 3 of which did not state an equivalence (DER) to dried ginger root. The unavailability of a DER for these interventions, as well as lack of subgroup analyses is a major limitation and contributor to variability of the included MA. The majority of studies did not clearly define clinical efficacy or report on the number of patients with a clinically relevant decrease in NVP symptoms. Additionally, many of the included MAs did not focus on the safety of ginger preparations and the included trials were not designed to capture differences in rare adverse effects between groups. Finally, only full-text publications in English could to be assessed in their entirety.

## Conclusions

This review summarizes the state of the evidence on ginger use during pregnancy and the maternal outcomes, which may respond to ginger use during pregnancy. Additionally, potential adverse effects are reported and summarized across study methodologies. No significant evidence was found supporting the use of a ginger intervention during pregnancy and lactation on lactation outcomes or treatment of gestational diabetes-related symptoms. But ginger interventions were found to have a significant beneficial effect on the symptoms of nausea, but not vomiting, of pregnancy when compared with placebo, despite the limitations in the available evidence. Ginger did not perform significantly better than the established NVP treatments including vitamin B6, which supports the evidence of the effectiveness of ginger in treating NVP. Additionally, there is no evidence of any significant adverse effects of ginger use during pregnancy across RCTs, as well as across observational studies of ginger use during pregnancy with a large number of participants. The limitations of this review are mainly the quality of the primary studies, the variety of ginger preparations used, as well as the large percent overlap of component RCTs between included MAs. This review highlights the need for high-quality RCTs that include multiple standardized-dosage ginger interventions with a large number of blinded diverse participants and predefined clinically significant outcomes to establish ginger as an effective treatment of NVP. Reporting the response rate of the treatment and comparator groups will also be useful in determining meaningful clinical efficacy. Additional RCTs with ginger preparations of verified dose and composition will allow for a rigorous and reproducible assessment of the safety and efficacy of ginger use in a pregnant population.

## Author contributions

The authors’ responsibilities were as follows – AJM, MSF, KAT: study design; MJF: search strategy design and implementation; KAT, CMA, JSD: study screening and data extraction; KAT, CMA: critical analyses; KAT: led analyses and drafting of the manuscript; KAT, CMA, MSF, MKS, RLB, PJS, AJM: contributed to interpretation of results, writing, and editing of the manuscript; and all authors: read and approved the final manuscript.

## Funding

This work was supported by the Bill and Melinda Gates Foundation (INV-047386). The supporting source identified ginger as a topic of interest but otherwise had no involvement in the analysis, manuscript writing and imposed no restrictions on publication.

## Data availability

Data described in the manuscript will be made available upon reasonable request.

## Conflict of interest

The authors report no conflicts of interest.
