# Supplementary material for: The Use of Ginger Bioactive Compounds in Pregnancy: An Evidence Scan and Umbrella Review of Existing Meta-Analyses
Source: Adv Nutr. 2024 Sep 28;15(11):100308. doi: 10.1016/j.advnut.2024.100308 (PMC11536023; doi:10.1016/j.advnut.2024.100308)
Supplement: Multimedia component 1 [file mmc1.pdf]

Supplementary Materials

The use of ginger bioactive compounds in pregnancy: an evidence scan and umbrella review of existing meta-analyses

Kendra A. Tiani[1], Cristina M. Arenaz[1], Maureen K. Spill[2], Margaret J. Foster[3], Julie S. Davis[2], Regan L. Bailey[1,5], Martha S. Field[4], Patrick J. Stover[5,6], Amanda J. MacFarlane[2,5*]

[1]Institute for Advancing Health Through Agriculture, Texas A&M University, College Station, TX, USA

[2]Texas A&M Agriculture, Food and Nutrition Evidence Center, Fort Worth, TX, USA

[3]Center for Systematic Reviews and Evidence Syntheses, Texas A&M University, College Station, TX, USA

[4]Division of Nutritional Sciences, Cornell University, Ithaca, NY, USA

[5]Department of Nutrition, Texas A&M University, College Station, TX, USA

[6]Department of Biochemistry and Biophysics, Texas A&M University, College Station, TX, USA

***Corresponding author:**
Amanda J. MacFarlane
Mailing Address: 500 Cherry Street, Suite 850, Fort Worth, Texas 76102
Telephone: 940-536-0148
amanda.macfarlane@ag.tamu.edu

PDF Table of Contents:

Supplementary Table S1. PRISMA-ScR checklist

Supplementary Table S2. Search strategy

Supplementary Table S3. References excluded during full-text evaluation

Supplementary Figure S1. AMSTAR-2 critical analysis

Supplementary Figure S2. CCA calculations for pairs of reviews

Excel Table of Contents:

Supplementary Table S4. Evidence scan extractions.

Supplementary Table S5. Umbrella review of meta-analysis primary studies

Supplementary Table S1. Preferred Reporting Items for Systematic reviews and Meta-Analyses extension for Scoping Reviews (PRISMA-ScR) checklist

SECTION	ITEM	PRISMA-ScR CHECKLIST ITEM	REPORTED ON PAGE #
**TITLE**			
Title	1	Identify the report as a scoping review.	1 (evidence scan and umbrella review)
**ABSTRACT**			
Structured summary	2	Provide a structured summary that includes (as applicable): background, objectives, eligibility criteria, sources of evidence, charting methods, results, and conclusions that relate to the review questions and objectives.	2
**INTRODUCTION**			
Rationale	3	Describe the rationale for the review in the context of what is already known. Explain why the review questions/objectives lend themselves to a scoping review approach.	5
Objectives	4	Provide an explicit statement of the questions and objectives being addressed with reference to their key elements (e.g., population or participants, concepts, and context) or other relevant key elements used to conceptualize the review questions and/or objectives.	5-6
**METHODS**			
Protocol and registration	5	Indicate whether a review protocol exists; state if and where it can be accessed (e.g., a Web address); and if available, provide registration information, including the registration number.	6
Eligibility criteria	6	Specify characteristics of the sources of evidence used as eligibility criteria (e.g., years considered, language, and publication status), and provide a rationale.	6-8
Information sources*	7	Describe all information sources in the search (e.g., databases with dates of coverage and contact with authors to identify additional sources), as well as the date the most recent search was executed.	6
Search	8	Present the full electronic search strategy for at least 1 database, including any limits used, such that it could be repeated.	6, Supplementary Table S2
Selection of sources of evidence†	9	State the process for selecting sources of evidence (i.e., screening and eligibility) included in the scoping review.	7-8, 10
Data charting process‡	10	Describe the methods of charting data from the included sources of evidence (e.g., calibrated forms or forms that have been tested by the team before their use, and whether data charting was done independently or in duplicate) and any processes for obtaining and confirming data from investigators.	8-11
Data items	11	List and define all variables for which data were sought and any assumptions and simplifications made.	8-11
Critical appraisal of individual	12	If done, provide a rationale for conducting a critical appraisal of included sources of evidence; describe	11

SECTION	ITEM	PRISMA-ScR CHECKLIST ITEM	REPORTED ON PAGE #
sources of evidence§		the methods used and how this information was used in any data synthesis (if appropriate).	
Synthesis of results	13	Describe the methods of handling and summarizing the data that were charted.	11-12
**RESULTS**			
Selection of sources of evidence	14	Give numbers of sources of evidence screened, assessed for eligibility, and included in the review, with reasons for exclusions at each stage, ideally using a flow diagram.	12-13, Fig. 1, Supplementary Table S3
Characteristics of sources of evidence	15	For each source of evidence, present characteristics for which data were charted and provide the citations.	13-18, Table 1, Figure 2, Table 2, Supplementary Tables S4 and S5
Critical appraisal within sources of evidence	16	If done, present data on critical appraisal of included sources of evidence (see item 12).	25-26, Supplementary Figure S1
Results of individual sources of evidence	17	For each included source of evidence, present the relevant data that were charted that relate to the review questions and objectives.	27-34, Table 3
Synthesis of results	18	Summarize and/or present the charting results as they relate to the review questions and objectives.	35-42, Figure 3, Supplementary Figure S2
**DISCUSSION**			
Summary of evidence	19	Summarize the main results (including an overview of concepts, themes, and types of evidence available), link to the review questions and objectives, and consider the relevance to key groups.	42-45
Limitations	20	Discuss the limitations of the scoping review process.	47-48
Conclusions	21	Provide a general interpretation of the results with respect to the review questions and objectives, as well as potential implications and/or next steps.	48-49
**FUNDING**			
Funding	22	Describe sources of funding for the included sources of evidence, as well as sources of funding for the scoping review. Describe the role of the funders of the scoping review.	50

JBI = Joanna Briggs Institute; PRISMA-ScR = Preferred Reporting Items for Systematic reviews and Meta-Analyses extension for Scoping Reviews.
* Where *sources of evidence* (see second footnote) are compiled from, such as bibliographic databases, social media platforms, and Web sites.
† A more inclusive/heterogeneous term used to account for the different types of evidence or data sources (e.g., quantitative and/or qualitative research, expert opinion, and policy documents) that may be eligible in a scoping review as opposed to only studies. This is not to be confused with *information sources* (see first footnote).
‡ The frameworks by Arksey and O'Malley (6) and Levac and colleagues (7) and the JBI guidance (4, 5) refer to the process of data extraction in a scoping review as data charting.
§ The process of systematically examining research evidence to assess its validity, results, and relevance before using it to inform a decision. This term is used for items 12 and 19 instead of "risk of bias" (which is more applicable to systematic reviews of interventions) to include and acknowledge the various sources of evidence that may be used in a scoping review (e.g., quantitative and/or qualitative research, expert opinion, and policy document).

From: Tricco AC, Lillie E, Zarin W, O'Brien KK, Colquhoun H, Levac D, et al. PRISMA Extension for Scoping Reviews (PRISMAScR): Checklist and Explanation. Ann Intern Med. 2018;169:467–473. doi: 10.7326/M18-0850.

Supplementary Table S2. Search strategy

Ginger Search Summary		Initial Search				Updated Search			
**Database**	**Interface**	**Date**	**Retrieved**	**Dups**	**Unique**	**Date**	**Retrieved**	**Dups**	**Unique**
Medline	Ovid	4/1/23	183	1	182	12/20/23	6	2	4
Embase	Ovid	4/2/23	166	78	88	12/20/23	16	6	10
Cab abstracts	Ovid	4/2/23	117	70	47	12/20/23	5	4	1
International Pharmaceutical Abstracts	Ovid	4/2/23	7	5	2	12/20/23	0	0	0
		Totals	473	154	319		27	12	15

Ovid MEDLINE(R) ALL <1946 to April 04, 2023>

1	exp ginger/ or exp ginger extract/	2005
2	(alpinetin or ginger).ti,ab,kw.	4262
3	(Alpinia blepharocalyx or Alnus firma or Zingiberaceae).ti,ab,kw.	1195
4	or/1-3	5531
5	exp Pregnancy/ or exp Pregnancy Complications/ or exp Pregnancy Outcome/	1031285
6	(prenatal* or postnatal* or antenatal* or maternal or pregnan*).ti,ab.	892747
7	exp Lactation/	47552
8	(lactat* or breastfeed* or nursing).ti,ab.	499883
9	exp Infant, Newborn/	669299
10	(infant* or baby or babies or newborn* or neonate*).ti,ab.	673487
11	or/5-10	2464983
12	(animals not humans).sh.	5076041
13	(mice or mouse or rat or rats).ti,ab.	2768073
14	in vitro study.ti,ab,kw.	35559
15	preclinical.ti,ab,kw.	126131
16	or/12-15	5976111
17	review.pt.	3133844
18	(medline or medlars or embase or pubmed or cochrane).tw,sh.	332002
19	(scisearch or psychinfo or psycinfo).tw,sh.	56930
20	(psychlit or psyclit).tw,sh.	918
21	cinahl.tw,sh.	43212
22	((hand adj2 search$) or (manual$ adj2 search$)).tw,sh.	16630
23	(electronic database$ or bibliographic database$ or computeri?ed database$ or online database$).tw,sh.	56663
24	(pooling or pooled or mantel haenszel).tw,sh.	143706
25	(peto or dersimonian or der simonian or fixed effect).tw,sh.	10462
26	(retraction of publication or retracted publication).pt.	27303
27	or/18-26	508475
28	17 and 27	216124
29	meta-analysis.pt.	178877
30	meta-analysis.sh.	178877
31	(meta-analys$ or meta analys$ or metaanalys$).tw,sh.	295063
32	(systematic$ adj5 review$).tw,sh.	319724
33	(systematic$ adj5 overview$).tw,sh.	3504
34	(quantitativ$ adj5 review$).tw,sh.	10399
35	(quantitativ$ adj5 overview$).tw,sh.	417
36	(quantitativ$ adj5 synthesis$).tw,sh.	4419
37	(methodologic$ adj5 review$).tw,sh.	8436
38	(methodologic$ adj5 overview$).tw,sh.	552
39	(integrative research review$ or research integration).tw.	174
40	or/29-39	472910
41	28 or 40	555605
42	randomized controlled trial.pt.	590348
43	(random$ or placebo$ or single blind$ or double blind$ or triple blind$).ti,ab.	1509338
44	(retraction of publication or retracted publication).pt.	27303
45	or/42-44	1638921
46	((comment or editorial or meta-analysis or practice-guideline or review or letter) not "randomized controlled trial").pt.	5368022
47	(random sampl$ or random digit$ or random effect$ or random survey or random regression).ti,ab. not "randomized controlled trial".pt.	122242
48	45 not (46 or 47)	1332834
49	exp cohort studies/	2466753
50	cohort$.tw.	834592
51	controlled clinical trial.pt.	95252
52	epidemiologic methods/	31613
53	limit 52 to yr=1966-1989	11199
54	or/49-51,53	2917268
55	exp case-control studies/	1405050
56	(case$ and control$).tw.	595812
57	or/55-56	1835992
58	(case$ and series).tw.	228888
59	case reports.pt.	2328125
60	(case$ adj2 report$).tw.	685432
61	(case$ adj2 stud$).tw.	307303
62	or/58-61	2913563
63	(ae or to or po or co).fs.	4426702
64	(safe or safety).ti,ab.	1003628
65	side effect$.ti,ab.	293019
66	((adverse or undesirable or harms$ or serious or toxic) adj3 (effect$ or reaction$ or event$ or outcome$)).ti,ab.	659126
67	exp product surveillance, postmarketing/	18077
68	exp adverse drug reaction reporting systems/	8760
69	exp clinical trials, phase iv/	374
70	exp poisoning/	168397
71	exp substance-related disorders/	308660
72	exp drug toxicity/	131019
73	exp abnormalities, drug induced/	14735
74	exp drug monitoring/	23463
75	exp drug hypersensitivity/	49472
76	(toxicity or complication$ or noxious or tolerability).ti,ab.	1595351
77	exp Postoperative Complications/	607616
78	exp Intraoperative Complications/	56586
79	or/63-78	6844736
80	(4 and 11) not 16	245
81	80 and 41	51
82	80 and 48	51
83	80 and 54	17
84	80 and 57	14
85	80 and 62	10
86	80 and 79	148
87	or/81-86	183

Embase <1974 to 2023 April 04>

1	exp ginger extract/ or exp ginger/	8874
2	(alpinetin or ginger or Alpinia blepharocalyx or Alnus firma or Zingiberaceae).ti,ab,kw.	7591
3	or/1-2	11869
4	exp pregnant woman/ or exp pregnancy complication/ or exp pregnancy outcome/	302436
5	(prenatal* or postnatal* or antenatal* or maternal or pregnan*).ti,ab.	1139035
6	exp lactation/	58326
7	(lactat* or breastfeed* or nursing).ti,ab.	580857
8	exp newborn/	597730
9	(infant* or baby or babies or newborn* or neonate*).ti,ab.	817023
10	or/4-9	2502884
11	exp animal experiment/	3069609
12	(animal$ not human$).sh,hw.	4821847
13	(mice or mouse or rat or rats).ti,ab.	3397351
14	in vitro study.ti,ab,kw.	41925
15	preclinical.ti,ab,kw.	193777
16	or/11-15	6302482
17	exp review/	3127382
18	(literature adj3 review$).ti,ab.	447430
19	exp meta analysis/	289590
20	exp "Systematic Review"/	428060
21	or/17-20	3483885
22	(medline or medlars or embase or pubmed or cinahl or amed or psychlit or psyclit or psychinfo or psycinfo or scisearch or cochrane).ti,ab.	448453
23	RETRACTED ARTICLE/	15041
24	or/22-23	463021
25	21 and 24	362311
26	(systematic$ adj2 (review$ or overview)).ti,ab.	384461
27	(meta?anal$ or meta anal$ or meta-anal$ or metaanal$ or metanal$).ti,ab.	352118
28	or/25-27	665934
29	(random$ or placebo$ or single blind$ or double blind$ or triple blind$).ti,ab.	2093102
30	RETRACTED ARTICLE/	15041
31	29 or 30	2107569
32	(book or conference paper or editorial or letter or review).pt. not exp randomized controlled trial/	5860865
33	(random sampl$ or random digit$ or random effect$ or random survey or random regression).ti,ab. not exp randomized controlled trial/	159529
34	31 not (32 or 33)	1789554
35	exp cohort analysis/	1014841
36	exp longitudinal study/	191334
37	exp prospective study/	864810
38	exp follow up/	2025218
39	cohort$.tw.	1446371
40	or/35-39	3885918
41	exp case control study/	222549
42	(case$ and control$).tw.	881444
43	or/41-42	949050
44	exp case study/	97815
45	(case$ and series).tw.	333692
46	case report/	2883862
47	(case$ adj2 report$).tw.	922747
48	(case$ adj2 stud$).tw.	411607
49	or/44-48	3660750
50	(ae or si or to or co).fs.	3518725
51	(safe or safety).ti,ab.	1543637
52	side effect$.ti,ab.	438090
53	((adverse or undesirable or harm$ or serious or toxic) adj3 (effect$ or reaction$ or event$ or outcome$)).ti,ab.	1054652
54	exp adverse drug reaction/	630966
55	exp drug toxicity/	159475
56	exp intoxication/	437152
57	exp drug safety/	553975
58	exp drug monitoring/	60347
59	exp drug hypersensitivity/	77622
60	exp postmarketing surveillance/	39082
61	exp drug surveillance program/	26694
62	exp phase iv clinical trial/	5381
63	exp drug surveillance program/	26694
64	exp phase iv clinical trial/	5381
65	(toxicity or complication$ or noxious or tolerability).ti,ab.	2350807
66	exp postoperative complication/	807260
67	exp Peroperative Complication/	59782
68	or/50-67	7431734
69	(3 and 10) not 16	525
70	69 and 27	28
71	69 and 34	92
72	69 and 40	51
73	69 and 43	21
74	69 and 49	18
75	69 and 67	0
76	or/70-75	166

CAB Abstracts <1910 to 2023 Week 13>

1	(Alpinetin or ginger or Alpinia blepharocalyx or Alnus firma or Zingiberaceae).ti,ab.	12265
2	(prenatal* or postnatal* or antenatal* or maternal or pregnan*).ti,ab.	240779
3	(lactat* or breastfeed* or nursing).ti,ab.	201528
4	(infant* or baby or babies or newborn* or neonate*).ti,ab.	150026
5	2 or 3 or 4	503409
6	(mice or mouse or rat or rats).ti,ab.	601387
7	in vitro study.ti,ab.	6661
8	preclinical.ti,ab.	8700
9	or/6-8	612626
10	review.ti,ab.	482536
11	(medline or medlars or embase or pubmed or cochrane).tw.	34264
12	(scisearch or psychinfo or psycinfo).tw.	2309
13	(psychlit or psyclit).tw.	11
14	cinahl.tw.	3520
15	((hand adj2 search$) or (manual$ adj2 search$)).tw.	1590
16	(electronic database$ or bibliographic database$ or computeri?ed database$ or online database$).tw.	8359
17	(pooling or pooled or mantel haenszel).tw.	38205
18	(peto or dersimonian or der simonian or fixed effect).tw.	2922
19	(retraction of publication or retracted publication).ti,ab.	1
20	or/11-19	74095
21	10 and 20	32436
22	meta-analysis.ti,ab.	29629
23	meta-analysis.ti,ab.	29629
24	(meta-analys$ or meta analys$ or metaanalys$).tw.	34718
25	(systematic$ adj5 review$).tw.	43202
26	(systematic$ adj5 overview$).tw.	626
27	(quantitativ$ adj5 review$).tw.	1814
28	(quantitativ$ adj5 overview$).tw.	140
29	(quantitativ$ adj5 synthesis$).tw.	908
30	(methodologic$ adj5 review$).tw.	1177
31	(methodologic$ adj5 overview$).tw.	116
32	(integrative research review$ or research integration).tw.	59
33	or/22-32	62398
34	21 or 33	70659
35	randomized controlled trial.ti,ab.	11816
36	(random$ or placebo$ or single blind$ or double blind$ or triple blind$).ti,ab.	506624
37	(retraction of publication or retracted publication).ti,ab.	1
38	or/35-37	506625
39	cohort$.tw.	85596
40	controlled clinical trial.ti,ab.	3275
41	or/39-40	88823
42	(case$ and control$).tw.	210624
43	(case$ and series).tw.	34756
44	case reports.ti,ab.	6049
45	(case$ adj2 report$).tw.	152157
46	(case$ adj2 stud$).tw.	211085
47	or/43-46	383314
48	(safe or safety).ti,ab.	243178
49	side effect$.ti,ab.	43023
50	((adverse or undesirable or harms$ or serious or toxic) adj3 (effect$ or reaction$ or event$ or outcome$)).ti,ab.	157263
51	(drug adj1 (monitor* or toxic* or hypersensit*)).ti,ab.	2133
52	(toxicity or complication$ or noxious or tolerability or poison*).ti,ab.	328506
53	((Postoperative or Intraoperative) adj2 Complication*).ti,ab.	3791
54	or/48-53	685942
55	(1 and 5) not 9	202
56	55 and 34	23
57	55 and 38	58
58	55 and 41	9
59	55 and 42	10
60	55 and 47	7
61	55 and 54	80
62	or/56-61	117

International Pharmaceutical Abstracts <1970 to March 2023>

1	(Alpinetin or ginger or Alpinia blepharocalyx or Alnus firma or Zingiberaceae).ti,ab.	527
2	(prenatal* or postnatal* or antenatal* or maternal or pregnan*).ti,ab.	12415
3	(lactat* or breastfeed* or nursing).ti,ab.	9596
4	(infant* or baby or babies or newborn* or neonate*).ti,ab.	11439
5	2 or 3 or 4	29275
6	(mice or mouse or rat or rats).ti,ab.	57400
7	in vitro study.ti,ab.	860
8	preclinical.ti,ab.	4186
9	or/6-8	61410
10	review.ti,ab.	71654
11	(medline or medlars or embase or pubmed or cochrane).tw.	10212
12	(scisearch or psychinfo or psycinfo).tw.	509
13	(psychlit or psyclit).tw.	20
14	cinahl.tw.	561
15	((hand adj2 search$) or (manual$ adj2 search$)).tw.	459
16	(electronic database$ or bibliographic database$ or computeri?ed database$ or online database$).tw.	1330
17	(pooling or pooled or mantel haenszel).tw.	3722
18	(peto or dersimonian or der simonian or fixed effect).tw.	216
19	(retraction of publication or retracted publication).ti,ab.	0
20	or/11-19	13798
21	10 and 20	8331
22	meta-analysis.ti,ab.	6514
23	meta-analysis.ti,ab.	6514
24	(meta-analys$ or meta analys$ or metaanalys$).tw.	7499
25	(systematic$ adj5 review$).tw.	7109
26	(systematic$ adj5 overview$).tw.	114
27	(quantitativ$ adj5 review$).tw.	137
28	(quantitativ$ adj5 overview$).tw.	12
29	(quantitativ$ adj5 synthesis$).tw.	53
30	(methodologic$ adj5 review$).tw.	107
31	(methodologic$ adj5 overview$).tw.	8
32	(integrative research review$ or research integration).tw.	3
33	or/22-32	11586
34	21 or 33	15638
35	randomized controlled trial.ti,ab.	3626
36	(random$ or placebo$ or single blind$ or double blind$ or triple blind$).ti,ab.	72934
37	(retraction of publication or retracted publication).ti,ab.	0
38	or/35-37	72934
39	cohort$.tw.	17034
40	controlled clinical trial.ti,ab.	1061
41	or/39-40	18073
42	(case$ and control$).tw.	11245
43	(case$ and series).tw.	2758
44	case reports.ti,ab.	3137
45	(case$ adj2 report$).tw.	14173
46	(case$ adj2 stud$).tw.	6660
47	or/43-46	22097
48	(safe or safety).ti,ab.	57807
49	side effect$.ti,ab.	24013
50	((adverse or undesirable or harms$ or serious or toxic) adj3 (effect$ or reaction$ or event$ or outcome$)).ti,ab.	55938
51	(drug adj1 (monitor* or toxic* or hypersensit*)).ti,ab.	4781
52	(toxicity or complication$ or noxious or tolerability or poison*).ti,ab.	52265
53	((Postoperative or Intraoperative) adj2 Complication*).ti,ab.	241
54	or/48-53	150220
55	(1 and 5) not 9	14
56	55 and 34	2
57	55 and 38	2
58	55 and 41	0
59	55 and 42	0
60	55 and 47	0
61	55 and 54	6
62	or/56-61	7

Supplementary Table S3. Excluded references

First author	Year	Title
**Exclusion reason: Wrong study design (n = 43)**		
Schrager	2023	The association of nausea and vomiting of pregnancy, its treatments, and select birth defects: findings from the national birth defect prevention study
Ahmed	2022	Irrational use of selected herbal medicines during pregnancy: a pharmacoepidemiological evidence from yemen
Jahan	2022	Use of herbal medicines during pregnancy in a group of Bangladeshi women
Belayneh	2022	A cross-sectional study of herbal medicine use and contributing factors among pregnant women on antenatal care follow-up at Dessie Referral Hospital, Northeast Ethiopia
Barnes	2022	Demographics, health literacy and health locus of control beliefs of Australian women who take complementary medicine products during pregnancy and breastfeeding: A cross-sectional, online, national survey
Ee	2022	Complementary medicines and therapies in clinical guidelines on pregnancy care: A systematic review
Gantner	2021	Use of herbal medicines for the treatment of mild mental disorders and/or symptoms during pregnancy: A cross-sectional survey
Schrager	2021	Trends in first-trimester nausea and vomiting of pregnancy and use of select treatments: Findings from the National Birth Defects Prevention Study
Dafam	2021	Use of herbal medicine during pregnancy and attitudes of pregnant women in Jos, Nigeria
El Hajj	2020	Herbal medicine use among pregnant women attending antenatal clinics in Lusaka Province, Zambia: A cross-sectional, multicentre study
Abd El-Mawla	2020	Prevalence and use of medicinal plants among pregnant women in Assiut governorate
Bhatia	2020	Complementary and alternative medicinal use amongst antenatal in a rural tertiary care hospital of Haryana
Peprah	2019	'We are nothing without herbs': a story of herbal remedies use during pregnancy in rural Ghana
Volqvartz	2019	Use of alternative medicine, ginger and licorice among Danish pregnant women - a prospective cohort study
Nega	2019	Medicinal plants and concomitant use with pharmaceutical drugs among pregnant women
Ahmed	2018	Herbal medicine use by pregnant women in Bangladesh: a cross-sectional study
Gilmartin	2018	Complementary medicines in pregnancy: recommendations and information sources of healthcare professionals in Australia
Dudi	2018	Medicinal plants used during traditional postnatal care practices in Rajasthan, India
Shawahna	2017	Which potential harms and benefits of using ginger in the management of nausea and vomiting of pregnancy should be addressed? a consensual study among pregnant women and gynecologists
Kissal	2017	Use of herbal product among pregnant women in Turkey
Birru	2017	Prevalence and associated factors of herbal medicine use among pregnant women on ANC follow-up at Gondar university referral hospital, Ethiopia: A cross-sectional study
Mekuria	2017	Prevalence and associated factors of herbal medicine use among pregnant women on antenatal care follow-up at University of Gondar referral and teaching hospital, Ethiopia: A cross-sectional study
Petersen	2015	Women's perception of risks of adverse fetal pregnancy outcomes: a large-scale multinational survey
John	2015	Herbal medicines use during pregnancy: A review from the Middle East
Gwak	2015	Study on prescriptions for pregnancy and childbirth of the royal family in Joseon dynasty
Elberry	2015	Evaluation of the use of non-prescribed medications and herbs by pregnant women
Chen	2014	Dietary changes during pregnancy and the postpartum period in Singaporean Chinese, Malay and Indian women: the GUSTO birth cohort study
Bayisa	2014	Use of herbal medicine among pregnant women on antenatal care at Nekemte hospital, Western Ethiopia
Kennedy	2013	Herbal medicine use in pregnancy: results of a multinational study
Sim	2013	The use of herbal medicines during breastfeeding: a population-based survey in Western Australia
Saadia	2013	Dietary practices of Saudi women during puerperium

Nordeng	2011	Use of herbal drugs during pregnancy among 600 Norwegian women in relation to concurrent use of conventional drugs and pregnancy outcome
Tamuno	2011	Use of herbal medicine among pregnant women attending a tertiary hospital in northern Nigeria
Broussard	2010	Herbal use before and during pregnancy
Louik	2010	Use of herbal treatments in pregnancy
Ensiyeh	2010	Zingiber officinale (ginger) might be better than vitamin B6 for treating nausea in pregnancy
Buckner	2005	Health food stores' recommendations for nausea and migraines during pregnancy
Refuerzo	2005	Use of over-the-counter medications and herbal remedies in pregnancy
Nordeng	2004	Use of herbal drugs in pregnancy: a survey among 400 Norwegian women
Hollyer	2002	The use of CAM by women suffering from nausea and vomiting during pregnancy
Ernst	2002	Health risks over the Internet: advice offered by "medical herbalists" to a pregnant woman
Tsui	2001	A survey of dietary supplement use during pregnancy at an academic medical center
Wilkinson	2000	What do we know about herbal morning sickness treatments? A literature survey
**Exclusion reason: Not in english (n = 28)**		
Najafabadi	2023	Comparison of the effects of ondansetron and ginger on nausea and vomiting in pregnancy: a clinical trial
Jitcharerntham	2022	The effects of using the Thai ways program to stimulate lactation on the amount and duration of milk flow in postpartum mothers
Jarineshin	2022	Non-pharmacological methods of controlling nausea and vomiting during pregnancy in Iran: a narrative review study
Sari	2021	Hand massage therapy and herbal drinks ginger honey for pregnant women with nausea vomiting
Cardoso	2019	[The use of phytotherapy during pregnancy: a global overview]
Monazzami	2019	The effect of ginger (Zingiber officinale) compression for treatment of breast engorgement on lactation self-efficacy
Sassanarakkit	2019	A study on instant ginger drink effect in enhancing postpartum breastfeeding in immediate post cesarean delivery
Monazzami	2019	The effect of hot ginger compress (Zingiber officinale) on the severity of breast engorgement in lactating women
Bager	2019	Safety of pregnant women ingesting extracts from the roots of ginger (Zingiber officinale) and the possible interaction between ginger and warfarin
Pakniat	2018	Comparison of the effect of chamomile, Ginger and vitamin B6 on treatment of nausea and vomiting in pregnancy: A randomized clinical trial
Nazari	2018	Comparison of the effects of ondansetron, Vitamin b6 and ginger rhizome in nausea and vomiting of pregnancy: A randomized clinical trial
Parsa	2017	A systematic on the medicinal plants used in the treatment of postpartum pain in Iran
Niazi	2017	Effect of education of pregnant women's bill of rights on midwives function and satisfaction of pregnant women referred to Imam Reza Hospital, Kaboudarahang, 2015
Dabirifard	2017	Frequency, causes and how to use medicinal herbs during pregnancy
Boltman-Binkowski	2016	A systematic review: Are herbal and homeopathic remedies used during pregnancy safe?
Jenett-Siems	2015	With ginger against nausea and vomiting: Asian root helps pregnant women better than placebo
KwakJinSook	2014	Systematic review of the effect of dried ginger powder on improvement of nausea and vomiting associated with early pregnancy or motion sickness
Pourmaleky	2013	Comparison between the effects of zintoma (Ginger) and mefenamic acid on after pain during postpartum in multiparous women
Narenji	2012	Comparison the effects of the ginger fresh root and vitamin B6 on the nausea and vomiting in pregnancy
Modares	2012	Effect of Ginger and Chamomile capsules on nausea and vomiting in pregnancy
Schulz	2010	Ginger against nausea and vomiting during pregnancy: Effective within 4 days in the framework of a randomized study with 67 participants
Sondergaard	2008	[Ginger, pregnancy nausea and possible fetal injuries (testosterone effect)]
Ben-Arye	2006	[Herbal medicine in womens' life cycle]
ZhenZhen	2006	Development of milk powder for pregnant women that could suppress the pregnancy related morning sickness, nausea, and vomiting
Betz	2005	[Is ginger a clinically relevant antiemetic? A systematic review of randomized controlled trials]
Neri	2000	Nonpharmacological treatment of hyperemesis gravidarum

Cai	1990	[Study on correction of abnormal fetal position by applying ginger paste at zhihying acupoint A. Report of 133 cases]
Lori	1972	[Food habits in Taiwan. 1. Food habits during the pregnant and lying-in period and the nursing method of the baby.]
**Exclusion reason: Not a full report (n = 11)**		
Lindblad	2016	Ginger for nausea and vomiting of pregnancy
ACOG	2015	Nausea and vomiting of pregnancy
Facchinetti	2011	How we might do without vitamins
Koren	2009	Use of natural products in pregnancy
Hoffman	2007	Ginger: an ancient remedy and modern miracle drug
JNMA	2006	Herbal medicine during pregnancy and lactation
Marcus	2005	Effectiveness and safety of ginger in the treatment of pregnancy-induced nausea and vomiting
Fugh-Berman	2005	Do no harm: avoidance of herbal medicines during pregnancy
Marcus	2005	Do no harm: avoidance of herbal medicines during pregnancy
Calvert	2005	Ginger: an essential oil for shortening labour?
Smith	2005	Ginger was equivalent to pyridoxine hydrochloride (vitamin B6) for reducing nausea and vomiting in pregnancy
**Exclusion reason: Wrong intervention (n = 10)**		
Zakarija-Grkovic	2020	Treatments for breast engorgement during lactation
Sridharan	2020	Interventions for treating hyperemesis gravidarum: a network meta-analysis of randomized clinical trials
Razmjouei	2020	Investigating the effect of non-pharmacological treatments on reduction of breast engorgement in breastfeeding women: a review study
Bumrungpert	2018	Effects of fenugreek, ginger, and turmeric supplementation on human milk volume and nutrient content in breastfeeding mothers: A randomized double-blind controlled trial
Tan	2018	Nausea and vomiting of pregnancy: Effects on quality of life and day-to-day function
Flake	2015	Practical selection of antiemetics in the ambulatory setting
Vlastarakos	2008	Treating common ear problems in pregnancy: what is safe?
Chuang	2007	Chinese herbal medicines used in pregnancy: A population-based survey in Taiwan
Glover	2003	Prescription, over-the-counter, and herbal medicine use in a rural, obstetric population
Mazzotta	1999	The perception of teratogenic risk by women with nausea and vomiting of pregnancy
**Exclusion reason: Wrong outcomes (n = 4)**		
Sayuti	2023	The pattern of herbal medicines use for breastfeeding mother in Jogonalan, Klaten, Indonesia: a mini survey
Adane	2020	Herbal medicine use and predictors among pregnant women attending antenatal care in Ethiopia: a systematic review and meta-analysis
McLay	2017	Pregnancy, prescription medicines and the potential risk of herb-drug interactions: a cross-sectional survey
Flake	2004	Practical selection of antiemetics.
**Exclusion reason: Wrong patient population (n = 3)**		
Lu	2022	The preventive and relieving effects of ginger on postoperative nausea and vomiting: A systematic review and meta-analysis of randomized controlled trials
Griffiths	2021	Interventions for preventing nausea and vomiting in women undergoing regional anaesthesia for caesarean section
Dabaghzadeh	2014	Ginger for prevention or treatment of drug-induced nausea and vomiting
**Exclusion reason: Not able to be retrieved or not available (n = 3)**		
Khabarov	2023	Nausea and vomiting of pregnancy: impact on quality of life
Behera	2017	Dietary assessment of tribal lactating mothers in Koraput district of Odisha
Biswas	2011	A single-masked, randomized, controlled trial of ginger extract in the treatment of nausea and vomiting of pregnancy

Supplementary Figure S1

A

	Review planning			Search, screening, extraction				Description	RoB & heterogeneity				MA method			CoI	
**Author Year**	**Item 1**	**Item 2***	**Item 3**	**Item 4***	**Item 5**	**Item 6**	**Item 7***	**Item 8**	**Item 9***	**Item 10**	**Item 13***	**Item 14**	**Item 11***	**Item 12**	**Item 15***	**Item 16**	**OVERALL**
**2023 Tan**	Y	Y	Y	P	Y	Y	N	Y	Y	N	Y	Y	Y	N	N	Y	**Critically Low**
**2022 Gaur**	Y	Y	Y	P	Y	Y	N	P	Y	N	Y	Y	Y	Y	Y	Y	**Low**
**2020 Hu**	Y	N	Y	P	Y	Y	N	P	Y	N	Y	Y	Y	Y	Y	Y	**Critically Low**
**2015 Matthews**	Y	Y	Y	Y	Y	Y	Y	P	Y	N	Y	Y	Y	N	N	Y	**Low**
**2014 Viljoen**	Y	Y	N	Y	Y	Y	N	P	Y	N	Y	Y	Y	N	N	Y	**Critically Low**
**2014 Thomson**	Y	N	Y	P	Y	N	N	P	Y	N	Y	Y	Y	N	N	Y	**Critically Low**
**2018 Sridharan**	Y	Y	Y	Y	Y	Y	N	Y	Y	N	Y	N	Y	Y	Y	Y	**Low**
***Critical Items**		*		*			*		*		*		*		*		

**Overall Rating:**	
**High**	1 non-critical item
**Moderate**	>1 non-critical item
**Low**	1 critical item
**Critically Low**	>1 critical item

B

**AMSTAR-2 Item No.**	
**1**	Did the research questions and inclusion criteria for the review include the components of PICO?
**2**	Did the report of the review contain an explicit statement that the review methods were established prior to the conduct of the review and did the report justify any significant deviations from the protocol?
**3**	Did the review authors explain their selection of the study designs for inclusion in the review?
**4**	Did the review authors use a comprehensive literature search strategy?
**5**	Did the review authors perform study selection in duplicate?
**6**	Did the review authors perform data extraction in duplicate?
**7**	Did the review authors provide a list of excluded studies and justify the exclusions?
**8**	Did the review authors describe the included studies in adequate detail?
**9**	Did the review authors use a satisfactory technique for assessing the risk of bias in individual studies that were included in the review?
**10**	Did the review authors report on the sources of funding for the studies included in the review?
**11**	If meta-analysis was performed, did the review authors use appropriate methods for statistical combination of results?
**12**	If meta-analysis was performed, did the review authors assess the potential impact of risk of bias in individual studies on the results of the meta-analysis or other evidence synthesis?
**13**	Did the review authors account for risk of bias in individual studies when interpreting/ discussing the results of the review?
**14**	Did the review authors provide a satisfactory explanation for, and discussion of, any heterogeneity observed in the results of the review?
**15**	If they performed quantitative synthesis, did the review authors carry out an adequate investigation of publication bias (small study bias) and discuss its likely impact on the results of the review?
**16**	Did the review authors report any potential sources of conflict of interest, including any funding they received for conducting the review?

Supplementary Figure S1. AMSTAR-2 critical analysis. *A)* Results of the AMSTAR-2 critical analysis by item and definition of overall ratings. *B)* AMSTAR-2 questionnaire item information. Abbreviations: CoI, conflict of interest; MA, meta-analysis; RoB, risk of bias; Y, yes; N, no; P, partial yes.

Supplementary Figure S2

	2023 Tan	2022 Gaur	2020 Hu	2018 Sridharan	2015 Matthews	2014 Viljoen
2022 Gaur	40%					
2020 Hu	69%	54%				
2018 Sridharan	74%	37%	60%			
2015 Matthews	50%	21%	45%	75%		
2014 Viljoen	53%	27%	56%	55%	65%	
2014 Thomson	25%	8%	46%	25%	29%	50%

Supplementary Figure S2. Corrected covered area calculations for pairs of reviews. The CCA was calculated for all cited primary studies between pairs of reviews. The majority of reviews had high levels of overlap of citations. Adapted from (70). CCA, corrected covered area.
